# Supplementary material for: A solvent-free processed low-temperature tolerant adhesive
Source: Nat Commun. 2024 Jun 12;15:5017. doi: 10.1038/s41467-024-49503-7 (PMC11169673; doi:10.1038/s41467-024-49503-7)
Supplement: Supplementary file 1 — Supplementary Information [file 41467_2024_49503_MOESM1_ESM.pdf]

Supplementary Information

A Solvent-free Processed Low-temperature Tolerant Adhesive

Xiaoming Xie[1,2], Yulian Jiang[2], Xiaoman Yao[1], Jiaqi Zhang[3], Zilin Zhang[2], Taoping Huang[1], Runhan Li[1]*, Yifa Chen[1]*, Shun-li Li[1], Ya-Qian Lan[1]*

1. School of Chemistry, South China Normal University, Guangzhou, 510006, P. R. China.

2. Department of Chemistry, Xinzhou Normal University, Xinzhou, Shanxi, 034000, China.

3. College of Physics and Optoelectronics, Taiyuan University of Technology, Taiyuan, 030024, China.

*Correspondence and requests for materials should be addressed to R. L (email: lirh949@m.scnu.edu.cn), Y. C (email: chyf927821@163.com) and Y. -Q. L. (email: yqlan@m.scnu.edu.cn).

1. Supplementary Notes

Note 1: Materials.

Note 2: Characterization.

Note 3: Computational Methods.

2. Supplementary Figures

Figure 1. The preparation of SSFP and PSFP adhesives.

Figure 2. FT-IR spectra of SSFP adhesive, SiW_{12}, and PEG.

Figure 3. PXRD pattern of the physical mixture of PEG and SiW_{12}.

Figure 4. Total XPS spectra of SSFP adhesive, SiW_{12} and PEG. a) SSFP adhesive. b) SiW_{12}. c) PEG.

Figure 5. XPS spectra of SSFP, PEG and SiW_{12}. a) XPS spectra of the C *1s* of SSFP and PEG. b) XPS spectra of the W *4f* of SSFP and SiW_{12}.

Figure 6. ^{13}C NMR spectra of SSFP and PEG.

Figure 7. SEM images of SSFP adhesive and contrast samples. a) SiW_{12}. b) PEG. c) PEG after heating at 90 ℃ for 2 h. d) SSFP adhesive.

Figure 8. Adhesion behaviors of the SSFP adhesive for adhering various substrates. a) SS. b) Ceramic. c) Rubber. d) Carnelian. e) Glass. f) Wood.

Figure 9. Comparison of the adhesion strengths with the reported POMs based adhesive. a) The total graph. b) The enlarge graph.

Figure 10. Adhesion strengths of SSFP adhesive on SS substrate during eight warming-cooling treating processes. The error bars represent mean ± standard deviation (n = 3 independent samples).

Figure 11. The distribution images of SSFP adhesive on SS substrate after detachment (the SSFP adhesive dispersed within the dashed line).

Figure 12. FT-IR spectra of the SSFP adhesives with different mass ratios (PEG : SiW_{12} = 2 : 5, 2 : 4, and 2 : 3).

Figure 13. ^{1}H NMR spectra of SSFP adhesives with different mass ratios (PEG : SiW_{12} = 2 : 5, 2 : 4, and 2 : 3).

Figure 14. PXRD patterns of SSFP adhesives with different mass ratios (PEG : SiW_{12} = 2 : 5, 2 : 4, and 2 : 3).

Figure 15. TGA curves of SSFP adhesives with different mass ratios (PEG : SiW_{12} = 2 : 5, 2 : 4, and 2 : 3).

Figure 16. Adhesion strengths of SSFP and PSFP adhesives with different mass ratios (PEG : POMs = 2 : 5, 2 : 4, and 2 : 3). The error bars represent mean ± standard deviation (n = 3 independent samples).

Figure 17. Digital photographs of the adhesives after heating at 90 ℃ for 2 h. a) PEG_{2k} and SiW_{12}. b) PEG_{4k} and SiW_{12}. c) PEG_{8k} and SiW_{12}. d) PEG_{10k} and SiW_{12}. e) PEG_{20k} and SiW_{12}.

Figure 18. Adhesion strengths of the different molecular weight of PEG based adhesives on SS substrate. The error bars represent mean ± standard deviation (n = 3 independent samples).

Figure 19. The digital photographs of SSAP adhesive.

Figure 20. Adhesion strengths of the SSAP and SSFP adhesives on SS substrate. The error bars represent mean ± standard deviation (n = 3 independent samples).

Figure 21. Characterization of the PSFP adhesive. a) ^{13}C NMR spectra of PSFP and PEG. b) ^{1}H NMR spectra of PSFP adhesives with different mass ratios (PEG : PW_{12} = 2 : 5, 2 : 4, and 2 : 3) and PEG. c) FT-IR spectra of PSFP adhesive, PW_{12}, and PEG. d) FT-IR spectra of PSFP adhesives with different mass ratios (PEG : PW_{12} = 2 : 5, 2 : 4, and 2 : 3). e) PXRD patterns of PSFP adhesive, physical mixture of PEG and PW_{12}, PW_{12}, and PEG. f) PXRD patterns of PSFP adhesives with different mass ratios (PEG : PW_{12} = 2 : 5, 2 : 4, and 2 : 3).

Figure 22. SEM images and elemental mapping of the samples. a) PW_{12}. b) PSFP adhesive. c) The corresponding elemental mapping.

Figure 23. TGA curves of the samples. a) PEG. b) PW_{12}. c-e) PSFP adhesives with different mass ratios (PEG : PW_{12} = 2 : 5, 2 : 4, and 2 : 3). (f) DSC spectra of PSFP adhesive (2 : 5), PW_{12}, and PEG.

Figure 24. Digital photographs of the samples. a) Mixture of PEG and $Na_3PW_{12}O_{40}$. b) Mixture of PEG and $Na_3PW_{12}O_{40}$ after heating at 90 ℃ for 2 h.

Figure 25. PXRD patterns of the samples. a) PEG. b) $Na_3PW_{12}O_{40}$. c) Physical mixture of PEG and $Na_3PW_{12}O_{40}$. d) Mixture of PEG and $Na_3PW_{12}O_{40}$ after heating at 90 ℃ for 2 h.

Figure 26. SEM images of the mixture for $Na_3PW_{12}O_{40}$ and PEG after heating, and the corresponding elemental mapping.

Figure 27. Digital photographs of the samples after heating at 90 ℃ for 2 h. a) Adhesive based on PCL and SiW_{12}. b) Mixture of PE and SiW_{12}. c) Mixture of PVDF and SiW_{12}.

Figure 28. Adhesion strengths of different polymers-based adhesives on SS substrate: PPG, PTMEG and PEG (Mn, ~2000). The error bars represent mean ± standard deviation (n = 3 independent samples).

Figure 29. Adhesion strengths of different polymers based adhesives on SS substrate: PCL and PEG (Mn, ~10000). The error bars represent mean ± standard deviation (n = 3 independent samples).

Figure 30. Digital photographs of the PEG analogues based adhesives. a) PEG. b) PEGME. c) PEGdME (Mn, ~2000).

Figure 31. Adhesion strengths of different polymers based adhesives on SS substrate: PEG, PEGME and PEGdME (Mn, ~2000). The error bars represent mean ± standard deviation (n = 3 independent samples).

Figure 32. Characterization of the different polymers based adhesives. a) Viscosity as a function of shear rate. b) Shear stress as a function of shear rate.

Figure 33. Photographs of the SSFP adhesive after immersion in organic solvents (*N*-hex) for 14 days.

Figure 34. Adhesion behavior of the SSFP adhesive in organic solvent. a) Stainless steel mould. b) Initial adhesion state. c) Lifted state (the adhesion area is 1.77 cm^2, and the volume of ethyl acetate is 500 mL).

Figure 35. Adhesion behavior of the solvent-free adhesives (EVA and SSFP). a) Photographs of the solvent-free adhesives soaking in mesitylene. b) Adhesion strength of the solvent-free adhesive adhered on SS substrate after soaking for 7 days. The error bars for **b** represent mean ± standard deviation (n = 3 independent samples).

Figure 36. Digital photographs of the adhesive after soaking in different solvents for 5 days. a) Water. b) THF. c) DMF.

Figure 37. The SiW_{12} leaching test of SSFP adhesive by soaking in water. a) UV-vis spectrum of SiW_{12} in water. b) The plot of absorbance change at 263 nm upon the concentration increase of SiW_{12}. c) The corresponding dissolution ratio of SiW_{12} in SSFP adhesive after soaking in water for different time.

Figure 38. Adhesion strengths of SSFP adhesive at different relative humidity. The error bars represent mean ± standard deviation (n = 3 independent samples).

Figure 39. Shear viscosity of the samples as a function of temperature. a) The SSFP adhesive. b) PEG.

Figure 40. DSC spectra of SSFP adhesive, SiW_{12}, and PEG.

Figure 41. TGA curves of SSFP adhesive, SiW_{12} and PEG. a) SSFP adhesive. b) SiW_{12}. c) PEG.

Figure 42. Photographs of the SSFP adhesive adhered in glass slices after treatment. a) 25 ℃. b) -196 ℃.

Figure 43. Photographs of the PEG adhered in glass slices at different temperature. a) 60 ℃. b) 25 ℃. c) -196 ℃.

Figure 44. Photographs of the EVA adhered in glass slices after treatment. a) 25 ℃. b) -196 ℃.

Figure 45. Adhesion strengths of SSFP adhesive on SS substrate at various temperatures. The error bars represent mean ± standard deviation (n = 3 independent samples).

Figure 46. Adhesion performance of SSFP adhesive in liquid nitrogen (-196 ℃). a) Adhesion strength of SSFP adhesive frozen for different times. b) The photograph of SSFP adhesive on SS after soaking in liquid nitrogen (-196 ℃) for 60 days. The error bars for **a** represent mean ± standard deviation (n = 3 independent samples).

Figure 47. Adhesion tests of the commercial hot melt glue EVA in liquid nitrogen. a) Initial adhesion state. b) Immersed state. c-d) Frost-cracked state.

Figure 48. Temperature-dependent FT-IR spectra of SSFP adhesive at different temperature conditions. a) $W{=}O_d$. b) $W–O_b–W$. c) $W–O_c–W$. d) C–O–C.

Figure 49. The radial distribution function (RDF) of POMs and PEGs for the production of 1 ns.

Figure 50. Independent gradient model based on Hirshfeld partition (IGMH) and interaction energies (ΔE, kJ/mol) between SiW_{12} and PEG for the adhesive formation. a) SiW_{12} and one PEGs. b) SiW_{12} and two PEGs. c) SiW_{12} and three PEGs.

Figure 51. The ratio of formative hydrogen bonds between PEG and SiW_{12} at 25 and -196 ℃ for the final 2 ns of the NVT simulation.

Figure 52. Snapshots of the aggregation behavior of PEG and SiW_{12} at 55 ℃ (the cubic boundaries are marked with black lines).

Figure 53. The interaction energy of PEG and SiW_{12} during cross-linking process at 25 and 55 ℃ for 2 ns of the NVT simulation.

Figure 54. The ratio of formative hydrogen bonds between PEG and SiW_{12} at 25 and 55 ℃ for the final 2 ns of the NVT simulation.

Figure 55. Snapshots of the aggregation behavior of PEG and PW_{12} at 25 ℃ (the cubic boundaries are marked with black lines).

Figure 56. The interaction energy of PEG and POMs (SiW_{12} and PW_{12}) during cross-linking process at 25 ℃ for 2 ns of the NVT simulation.

Figure 57. The ratio of formative hydrogen bonds between PEG and POMs (SiW_{12} and PW_{12}) at 25 ℃ for the final 2 ns of the NVT simulation.

Figure 58. Adhesive mechanism and MD simulation based on the NPT ensemble. a) MD simulations of configurations of molecular models of SSFP and SS substrate. b) The interaction energy and the ratio of formative hydrogen bonds between SSFP adhesive and SS substrate at-196, 25, and 55 ℃. c) The comparison of interaction energy and the ratio of formative hydrogen bonds between SSFP and PSFP adhesives on SS substrate.

3. Supplementary Tables

Table 1. Performance comparison of the reported POMs based adhesives, and the test method is the same in the all references (lap joint).

Table 2. Comparison of representative solvent-free adhesives in terms of their adhesion strength, low temperature tolerance and temperature tolerance range.

Supplementary Notes

Supplementary Note 1: Materials.

All solvents and reagents obtained from commercial sources were used without further purification. PEG (Mn, ~2000 and ~10000 Da, 98%), PEGME (Mn, ~2000 Da, 96%), and PEGdME (Mn, ~2000 Da, 96%) were bought from Aladdin Industrial Corporation. PPG (Mn, ~2000 Da, 95%), PTMEG (Mn, ~2000 Da, 96%), PCL (Mn, ~10000 Da, 96%), PE (Mn, ~400000 Da, 95%), and PVDF (Mn, ~180000 Da, 97%) were purchased from Macklin Biochemical Technology Co., Ltd. (Shanghai). SiW_{12} (99%), PW_{12} (99%), $Na_3PW_{12}O_{40}$ (99%), mesitylene, isopropyl ether, ethyl acetate, octanoic acid, petroleum ether, n-hexane, dioxane, and 1,4-dibromobutane were obtained from Shanghai Titan Scientific Co., Ltd. The EVA was bought from the Taobao store.

Supplementary Note 2: Characterization.

Powder X-ray diffraction (PXRD) patterns were recorded on a Rigaku SmartLab diffractometer (Rigaku SmartLab, Japan) with Cu Kαradiation at 45 kV, 200 mA. Morphological and structural characterizations were performed by a field-emission scanning electron microscope (SEM, Hitachi SU-8010) system and energy-dispersive spectral (EDS) analysis instrument. X-ray photoelectron spectroscopy (XPS) measurements were conducted by a Thermo Fisher Scientific Escalab 250 Xi. Fourier transform infrared spectrometry (FT-IR) spectra were tested using a Bruker Optics Tensor 27 in the range of 4000-600 cm^{-1} with KBr pellets. Solid-state ^{13}C CP/MAS NMR was performed on a Bruker Avance NEO 400 WB spectrometer. ^{1}H NMR and ^{13}C NMR spectra were collected on a Bruker AVANCE 500 MHz spectrometer. ^{32}Si NMR spectra were collected on a Bruker AVANCE 500 MHz spectrometer (10 s for relaxation time, 3000 scans). Thermogravimetric analysis (TGA) was tested on a TGA5500 using a heating rate of 10 ℃ min^{-1} from 30 to 800 ℃ in nitrogen atmosphere. Differential Scanning Calorimeter (DSC) measurements were performed by a TAQ200 with a heating rate of 10 ℃ min^{-1} from -60 to 60 ℃ in nitrogen atmosphere. All lap shear tests were tested by a universal testing machine at a strain rate of 10 mm min^{-1} at ambient temperature. The cycling test used cycles of lap shearing and interfacial adhesion. Rheological studies were collected on a Rotary rheometer (Austria Anton Paar, MCR302). Degree of crosslinking were performed on low field NMR analyzer (MesoMR23/12-060H-I).

Supplementary Note 3: Computational details.

Density functional theory (DFT) calculations

Firstly, PEG fragments with five polymerization degrees are selected as the PEG computational model. The interaction energy calculations between the POMs and PEGs are performed by DFT simulation with Gaussian 09 program[1] at the B3LYP-D3[2] functional. The 6-31 +G** basis set is used for H atom involved in the POMs.The LanL2DZ basis sets[3] are used for the W atoms, and 6-31G* basis sets are employed for the other main-group atoms. The independent gradient model based on Hirshfeld (IMGH) partition is performed using Multiwfn[4]. Images of the 3D structures are prepared with VMD visualization program[5].

Interaction energies have been evaluated with the following formula:

$$E_{Int} = E_{POM\text{-}nPEG} - (E_{POM} + n*E_{PEG})$$

Where, E_{POM} is the energy of the POM structure alone, E_{PEG} is the energy of the PEG polymer (n = 1, 2, 3), and $E_{POM\text{-}nPEG}$ is the total energy of POM-nPEG complexes.

Molecule dynamics (MD) simulations

The structures of MD simulation are consist of 30 $H_4SiW_{12}O_{40}$ or 30 $H_3PW_{12}O_{40}$, and 150 PEG fragments (POM : PEG = 1 : 5). The models for POM and PEG can be obtained at ground-state geometric structures of DFT calculations, and the RESP charge[6] of POMs and PEGs are obtained with Multiwfn at same theoretical level. The stainless-steel plate is built with 3000 chromium oxide, 270 nickel oxide and 450 manganese oxide based on the components of the 2Cr13 stainless steel used in the test. The mix molecules are placed in 8 × 8 × 12 nm^3 simulation box, respectively.

The GROMACS 2018.8 simulation package[6-8] is employed for the MD simulations. The Lennard-Jones (LJ) potential is used to describe the van der Waals force, and the electriccharge is used to describe the electrostatic force, etc. The H, C, O, and P atoms are used in OPLS force field,[9] and Si, W, Ni, Cr, Mn atoms are chosen from UFF force field.[10] The cutoff distance for short-range nonbonded interactions is set to be 18 Å. The V-rescale temperature coupling method[11] is used to maintain three temperature levels at 77.15, 298.15 and 328.15 K, respectively. The three-dimensional periodic boundary conditions (PBC) have been applied for all of the MD simulations. After energy minimization of the box, two cycles of annealing are carried out in the equilibrate the system with velocity-rescale thermostat and Berendsen barostat. To obtain the generated phase of the global minimum configuration, a further 2 ns production simulations are carried out with parrinello-rahman barostat. Furthermore, the NVT ensemble of POM and PEG in 4 × 4 × 4 nm^3 simulation box has also be considered with running over 2 ns in steps of 2 fs. After simulation run, the interaction energy and hydrogen bond percentage between POMs and PEGs are analyzed. Snapshots and movies of the aggregation behavior of are prepared with VMD visualization program.

Supplementary Figures

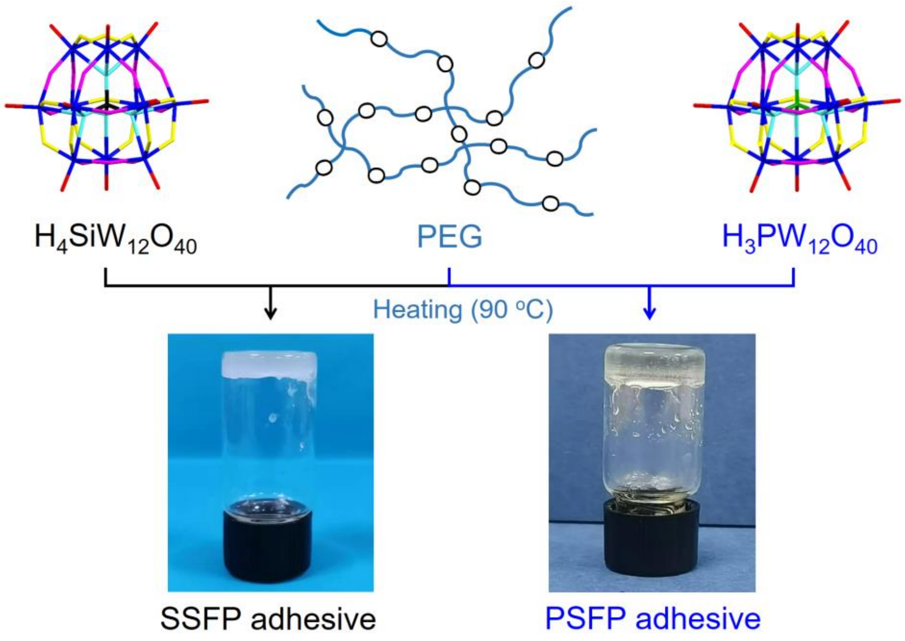

Supplementary Figure 1. The preparation of SSFP and PSFP adhesives.

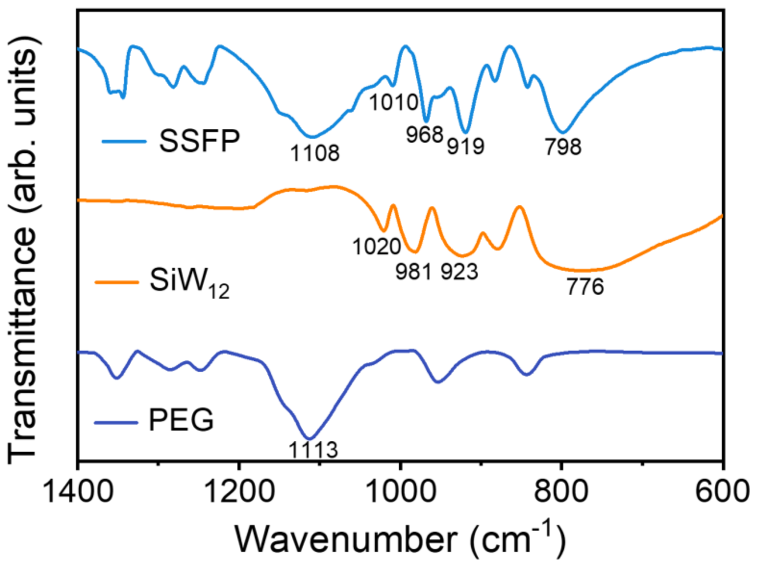

Supplementary Figure 2. FT-IR spectra of SSFP adhesive, SiW_{12}, and PEG.

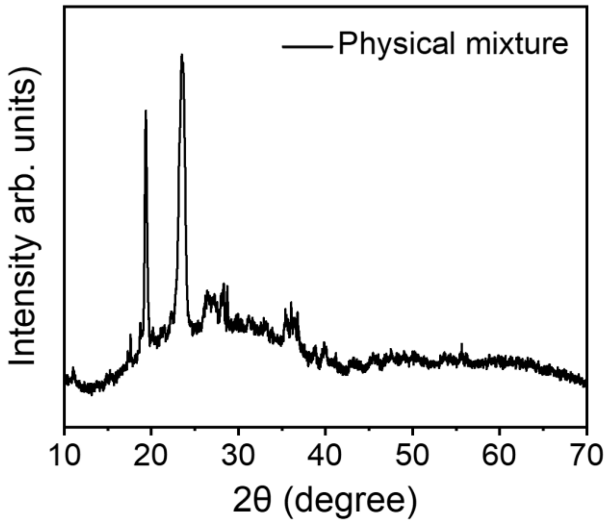

Supplementary Figure 3. PXRD pattern of the physical mixture of PEG and SiW_{12}.

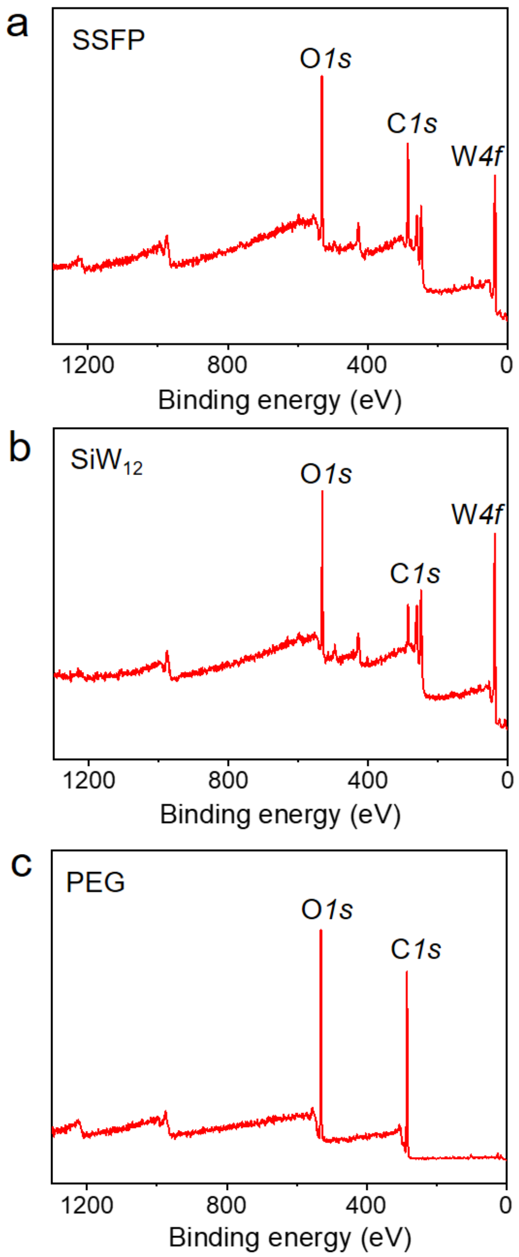

Supplementary Figure 4. Total XPS spectra of SSFP adhesive, SiW_{12} and PEG. a) SSFP adhesive. b) SiW_{12}. c) PEG.

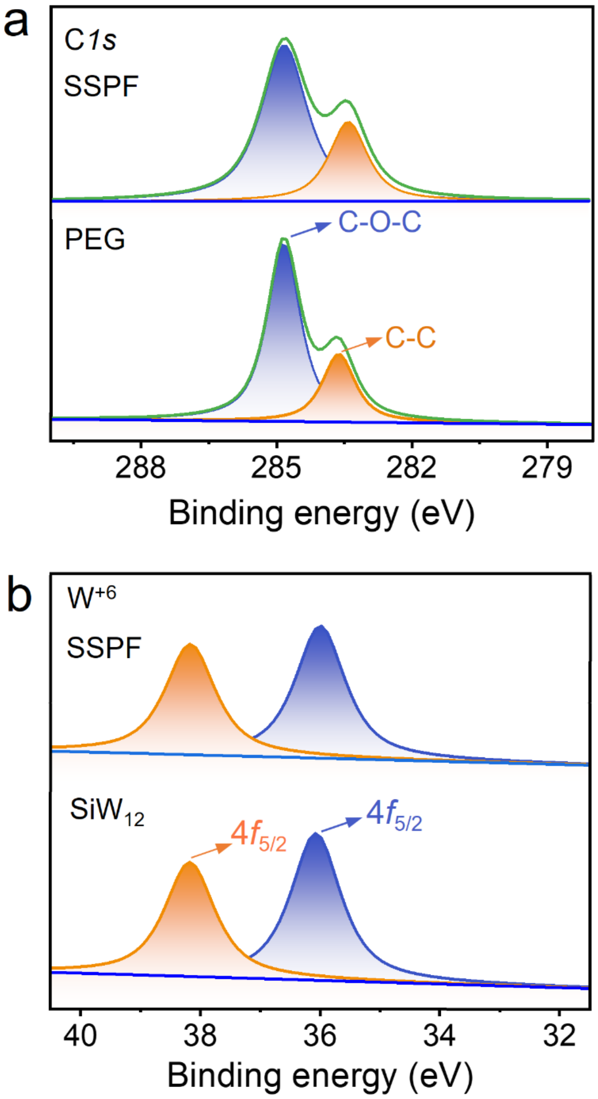

Supplementary Figure 5. XPS spectra of SSFP, PEG and SiW_{12}. a) XPS spectra of the C*1s* of SSFP and PEG. b) XPS spectra of the W*4f* of SSFP and SiW_{12}.

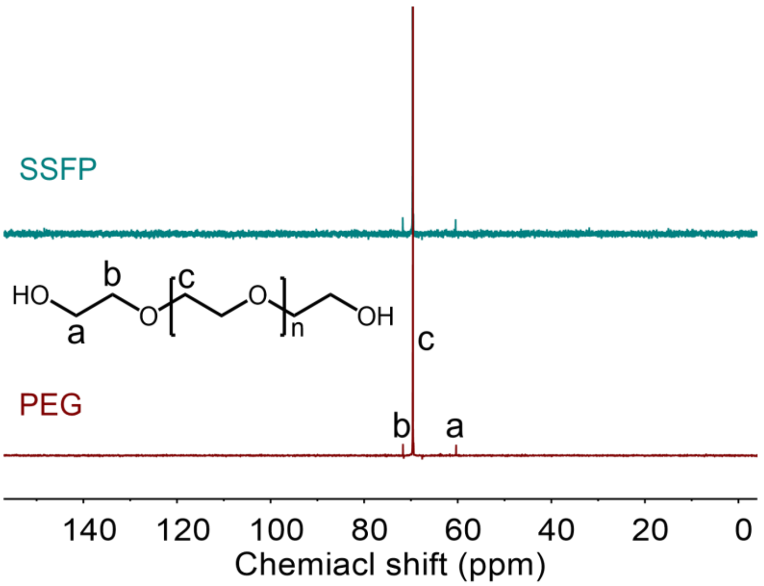

Supplementary Figure 6. ^{13}C NMR spectra of SSFP and PEG.

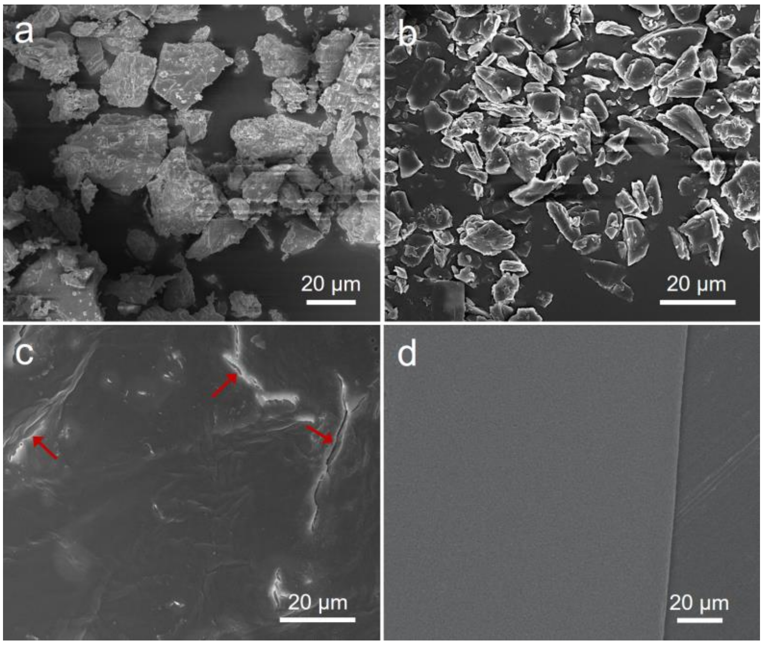

Supplementary Figure 7. SEM images of SSFP adhesive and contrast samples. a) SiW_{12}. b) PEG. c) PEG after heating at 90 ℃ for 2 h. d) SSFP adhesive.

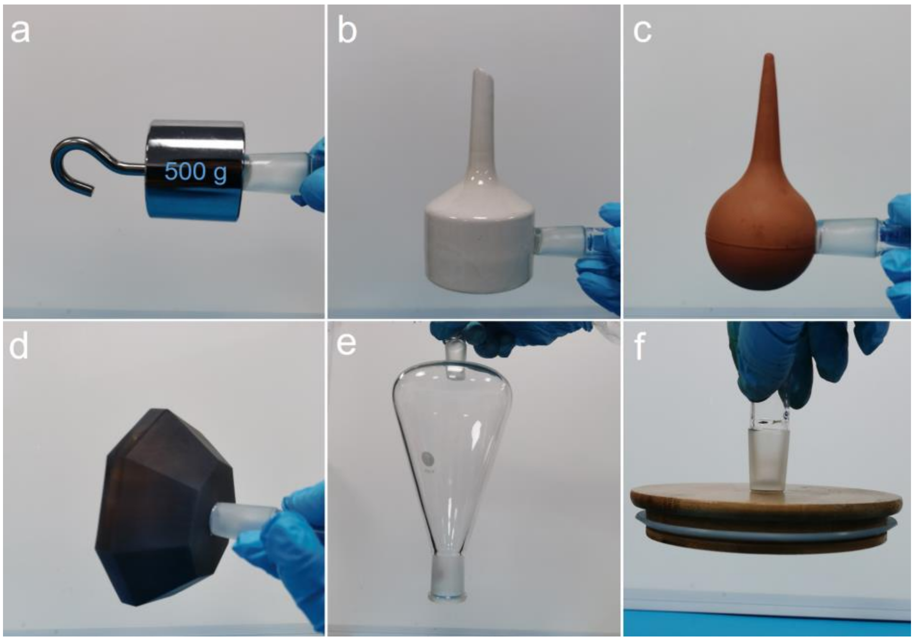

Supplementary Figure 8. Adhesion behaviors of the SSFP adhesive for adhering various substrates. a) SS. b) Ceramic. c) Rubber. d) Carnelian. e) Glass. f) Wood.

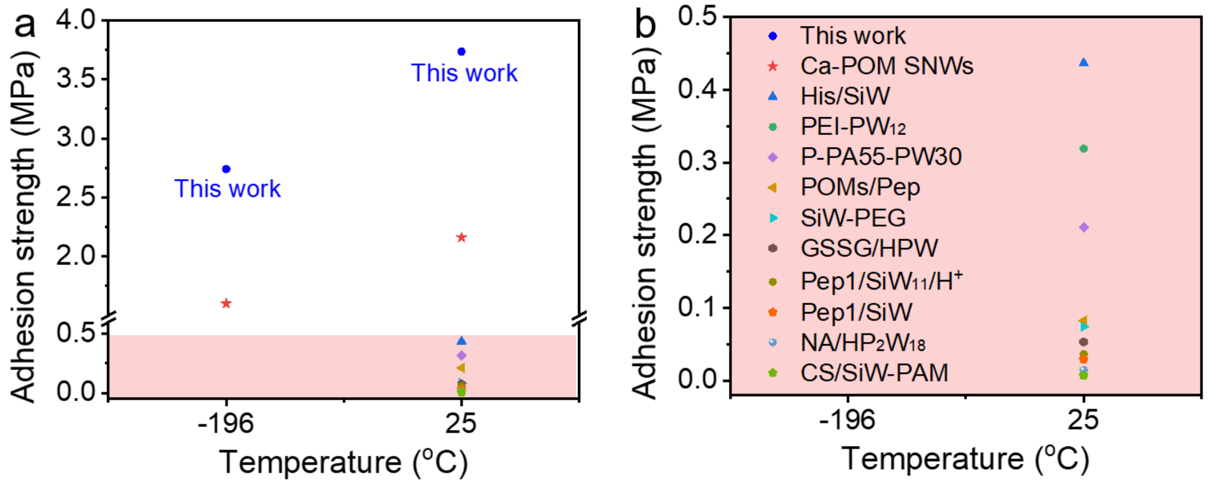

Supplementary Figure 9. Comparison of adhesion strengths with the reported POMs based adhesive[12-22]. a) The total graph. b) The enlarge graph.

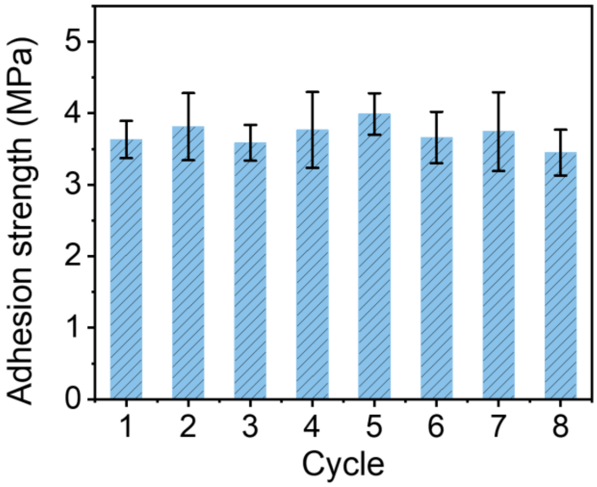

Supplementary Figure 10. Adhesion strengths of SSFP adhesive on SS substrate during eight warming-cooling treating processes. The error bars represent mean ± standard deviation (n = 3 independent samples).

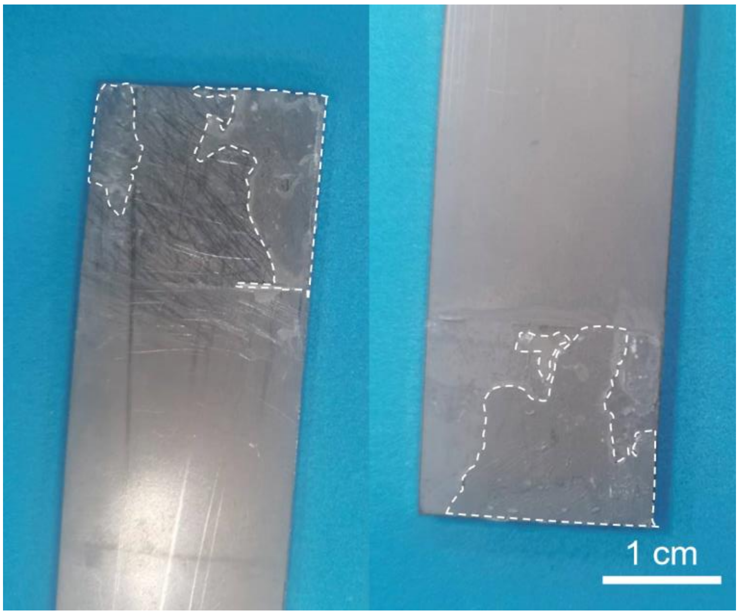

Supplementary Figure 11. The distribution images of SSFP adhesive on SS substrate after detachment (the SSFP adhesive dispersed within the dashed line).

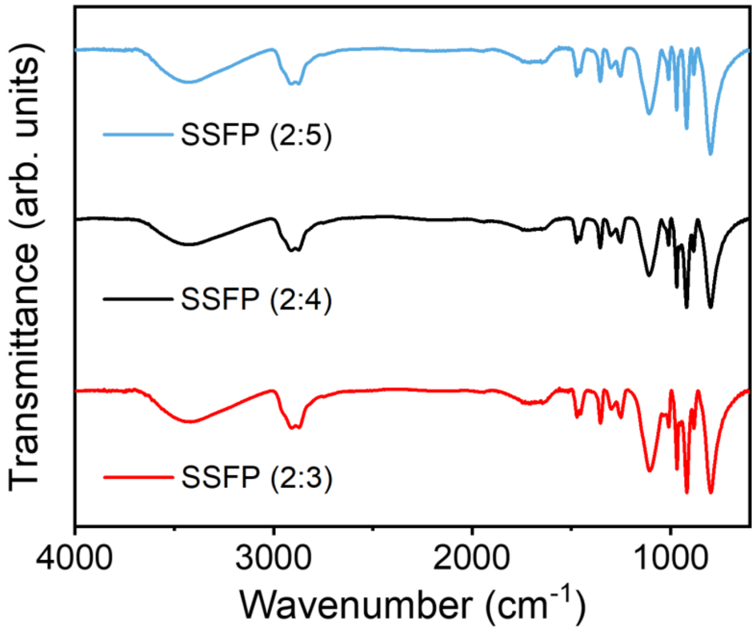

Supplementary Figure 12. FT-IR spectra of the SSFP adhesives with different mass ratios (PEG : SiW_{12} = 2 : 5, 2 : 4, and 2 : 3).

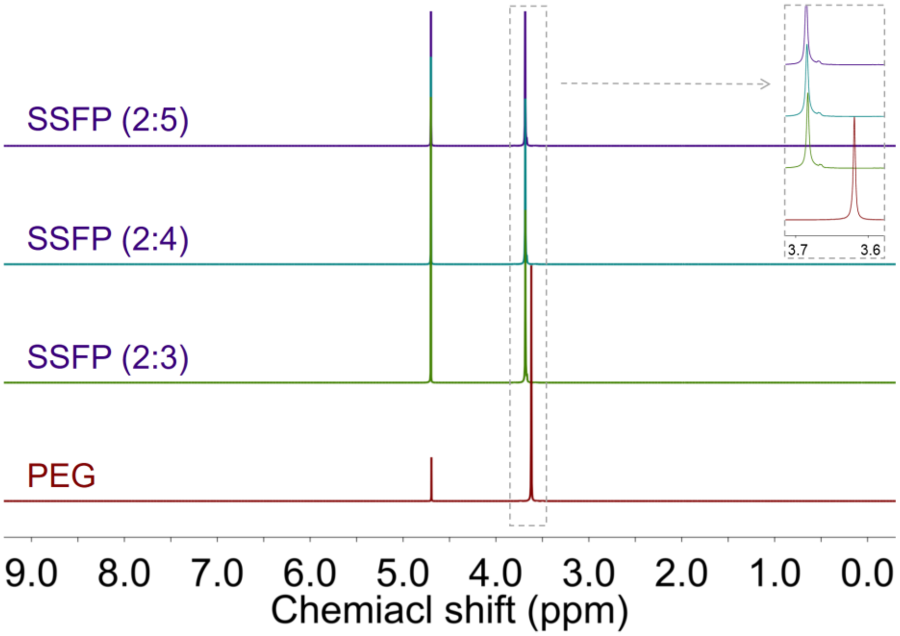

Supplementary Figure 13. ^{1}H NMR spectra of SSFP adhesives with different mass ratios (PEG : SiW_{12} = 2 : 5, 2 : 4, and 2 : 3).

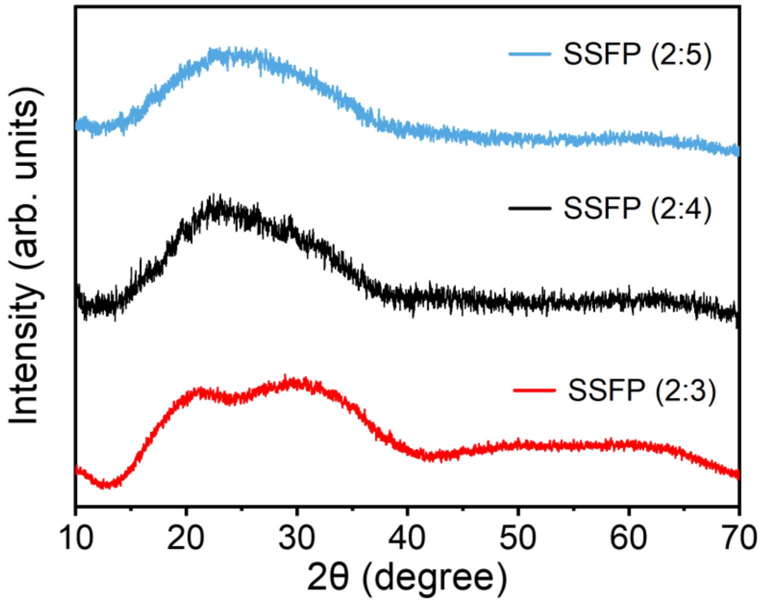

Supplementary Figure 14. PXRD patterns of SSFP adhesives with different mass ratios (PEG : SiW_{12} = 2 : 5, 2 : 4, and 2 : 3).

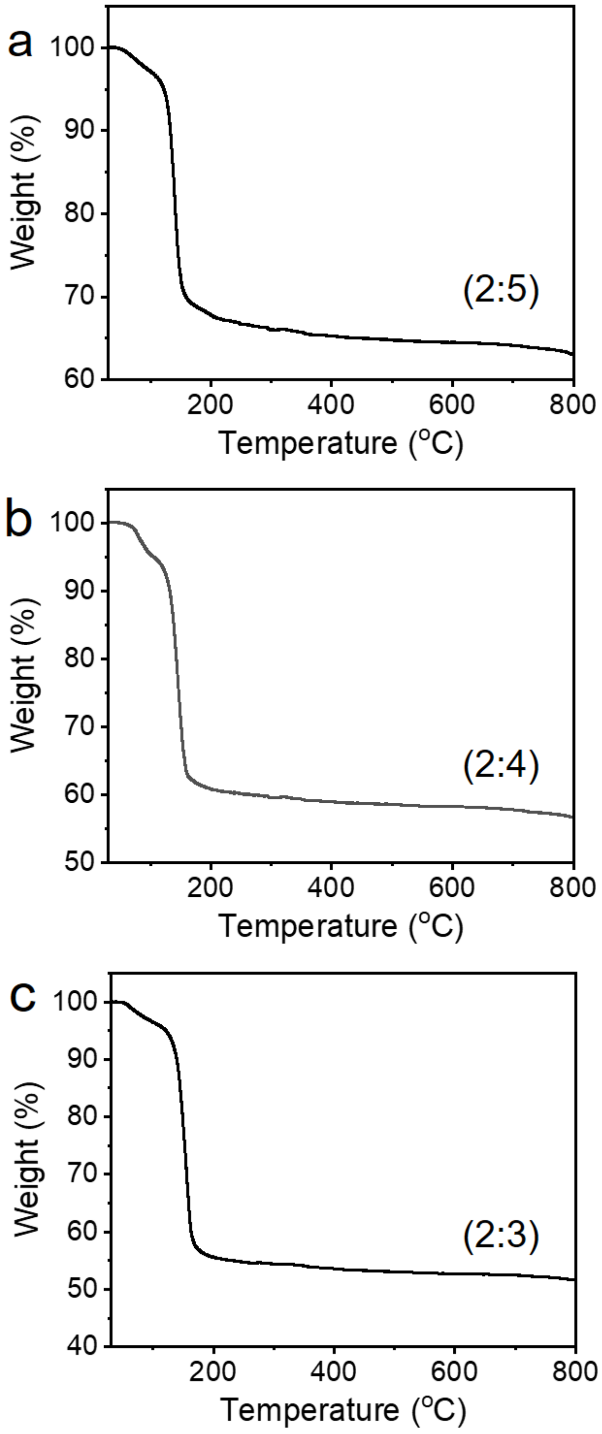

Supplementary Figure 15. TGA curves of SSFP adhesives with different mass ratios (PEG : SiW_{12} = 2 : 5, 2 : 4, and 2 : 3).

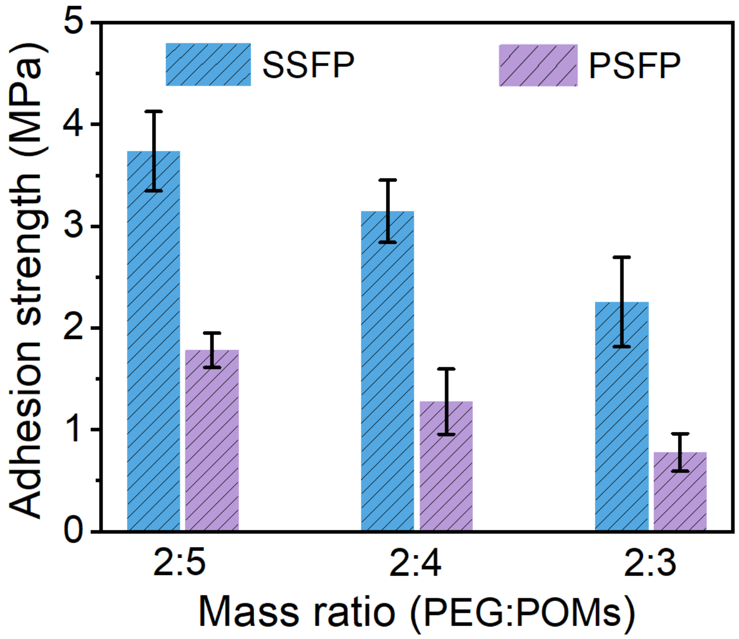

Supplementary Figure 16. Adhesion strengths of SSFP and PSFP adhesives with different mass ratios (PEG : POMs = 2 : 5, 2 : 4, and 2 : 3). The error bars represent mean ± standard deviation (n = 3 independent samples).

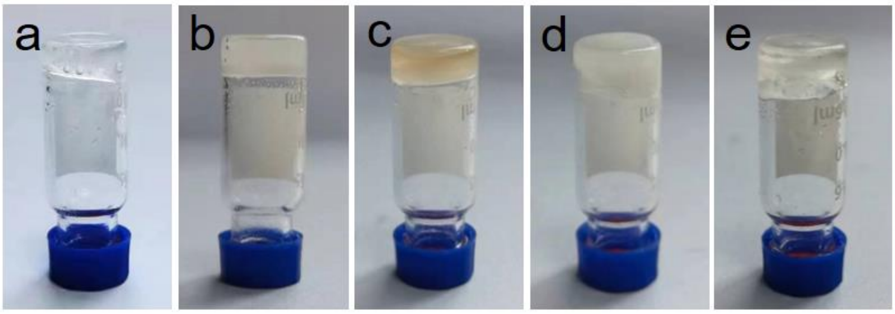

Supplementary Figure 17. Digital photographs of the adhesives after heating at 90 ℃ for 2 h. a) PEG_{2k} and SiW_{12}. b) PEG_{4k} and SiW_{12}. c) PEG_{8k} and SiW_{12}. d) PEG_{10k} and SiW_{12}. e) PEG_{20k} and SiW_{12}.

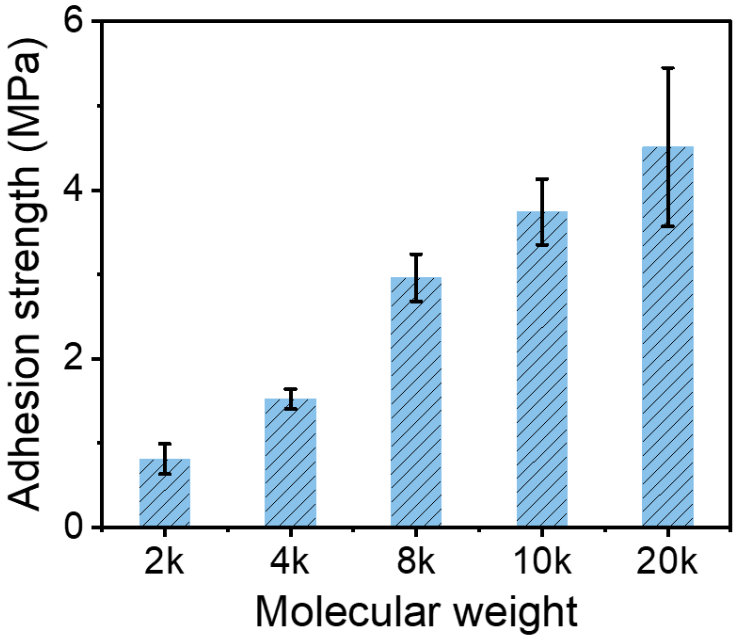

Supplementary Figure 18. Adhesion strengths of the different molecular weight of PEG based adhesives on SS substrate. The error bars represent mean ± standard deviation (n = 3 independent samples).

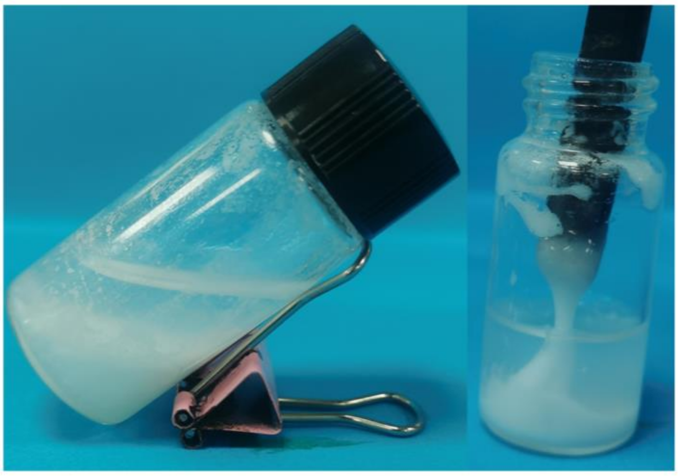

Supplementary Figure 19. The digital photographs of SSAP adhesive.

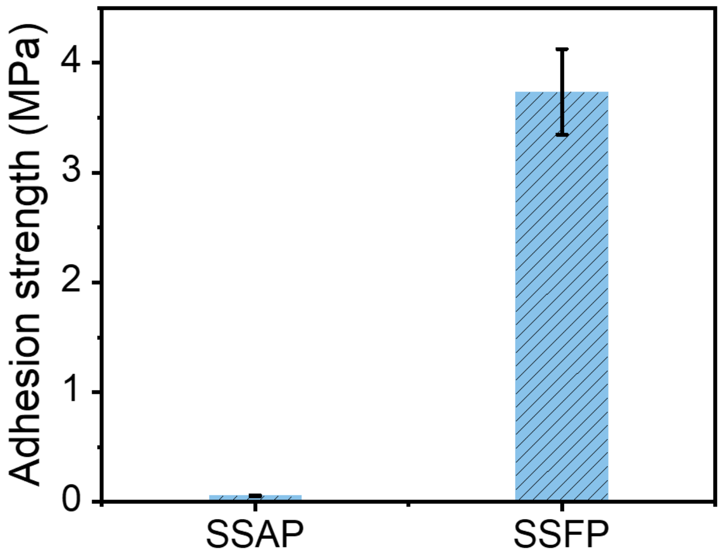

Supplementary Figure 20. Adhesion strengths of the SSAP and SSFP adhesives on SS substrate. The error bars represent mean ± standard deviation (n = 3 independent samples).

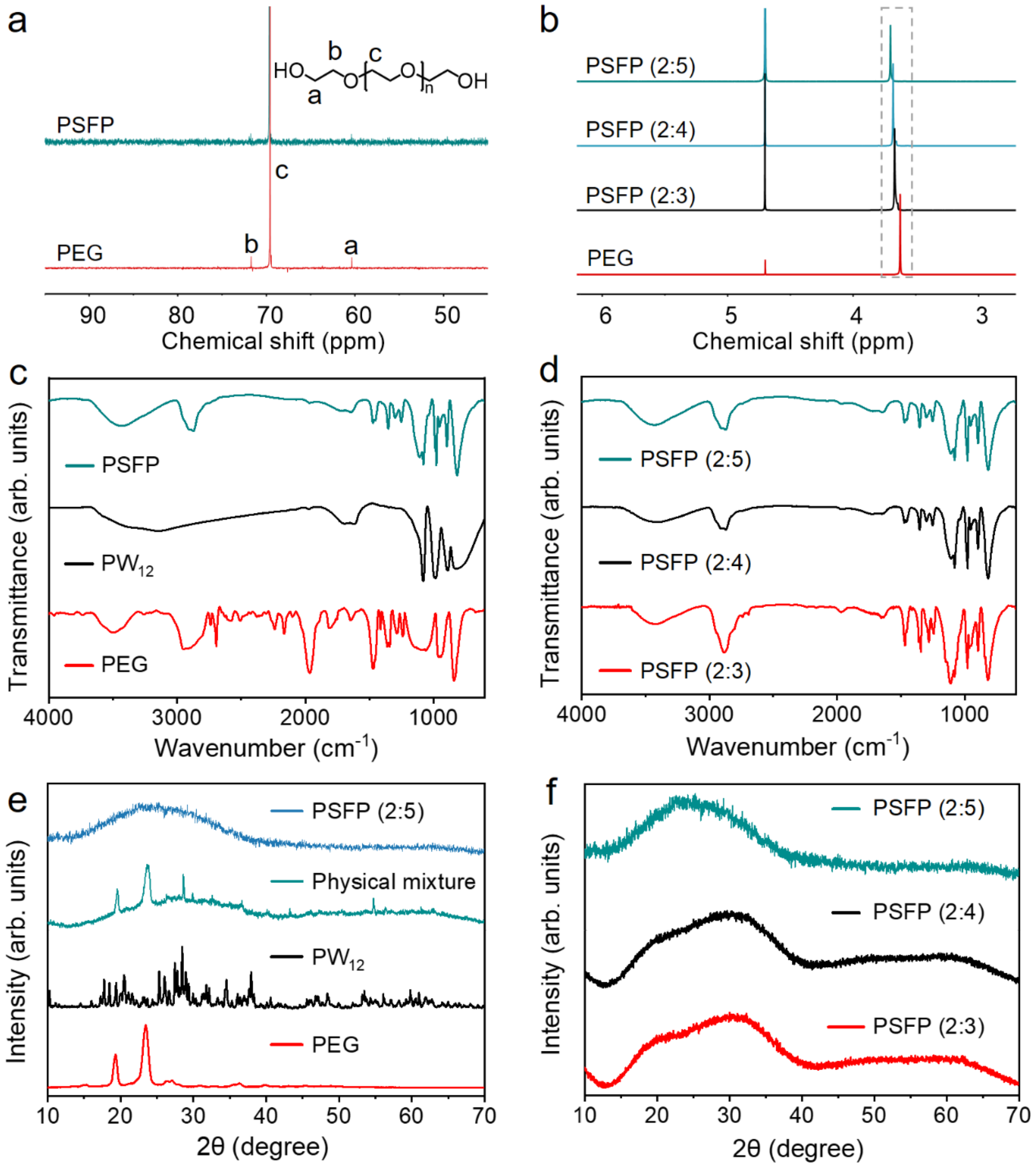

Supplementary Figure 21. Characterization of the PSFP adhesive. a) ^{13}C NMR spectra of PSFP and PEG. b) ^{1}H NMR spectra of PSFP adhesives with different mass ratios (PEG : PW_{12} = 2 : 5, 2 : 4, and 2 : 3) and PEG. c) FT-IR spectra of PSFP adhesive, PW_{12}, and PEG. d) FT-IR spectra of PSFP adhesives with different mass ratios (PEG : PW_{12} = 2 : 5, 2 : 4, and 2 : 3). e) PXRD patterns of PSFP adhesive, physical mixture of PEG and PW_{12}, PW_{12}, and PEG. f) PXRD patterns of PSFP adhesives with different mass ratios (PEG : PW_{12} = 2 : 5, 2 : 4, and 2 : 3).

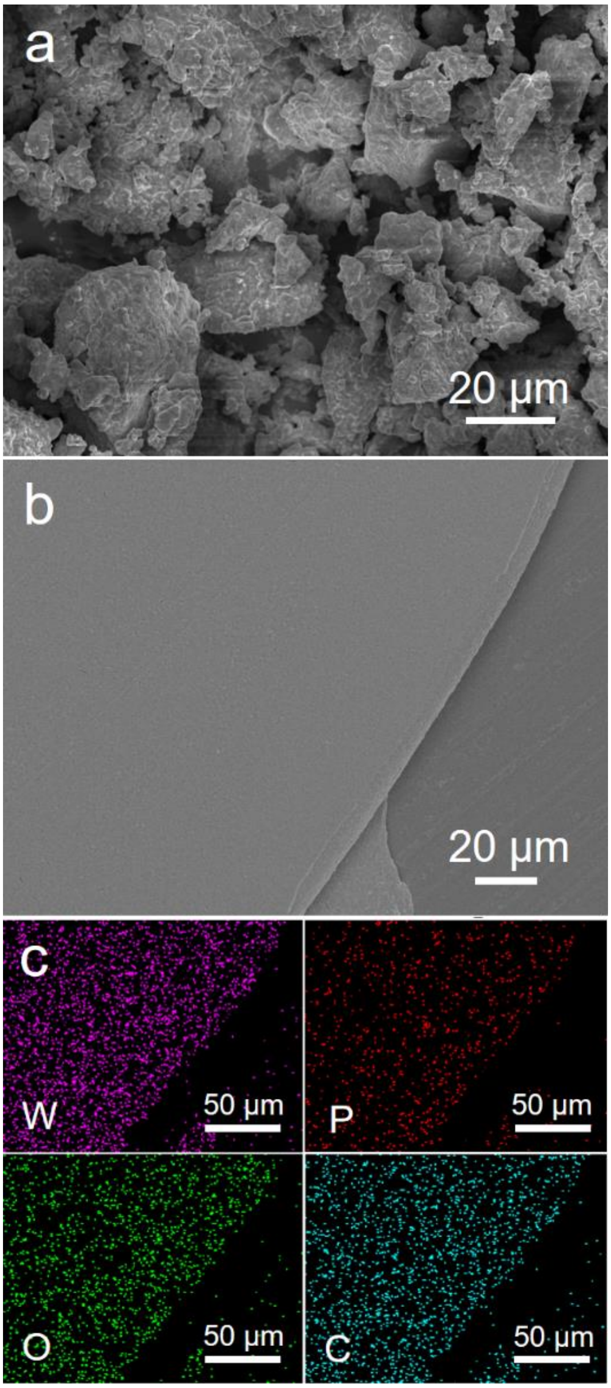

Supplementary Figure 22. SEM images and elemental mapping of the samples. a) PW_{12}. b) PSFP adhesive. c) The corresponding elemental mapping.

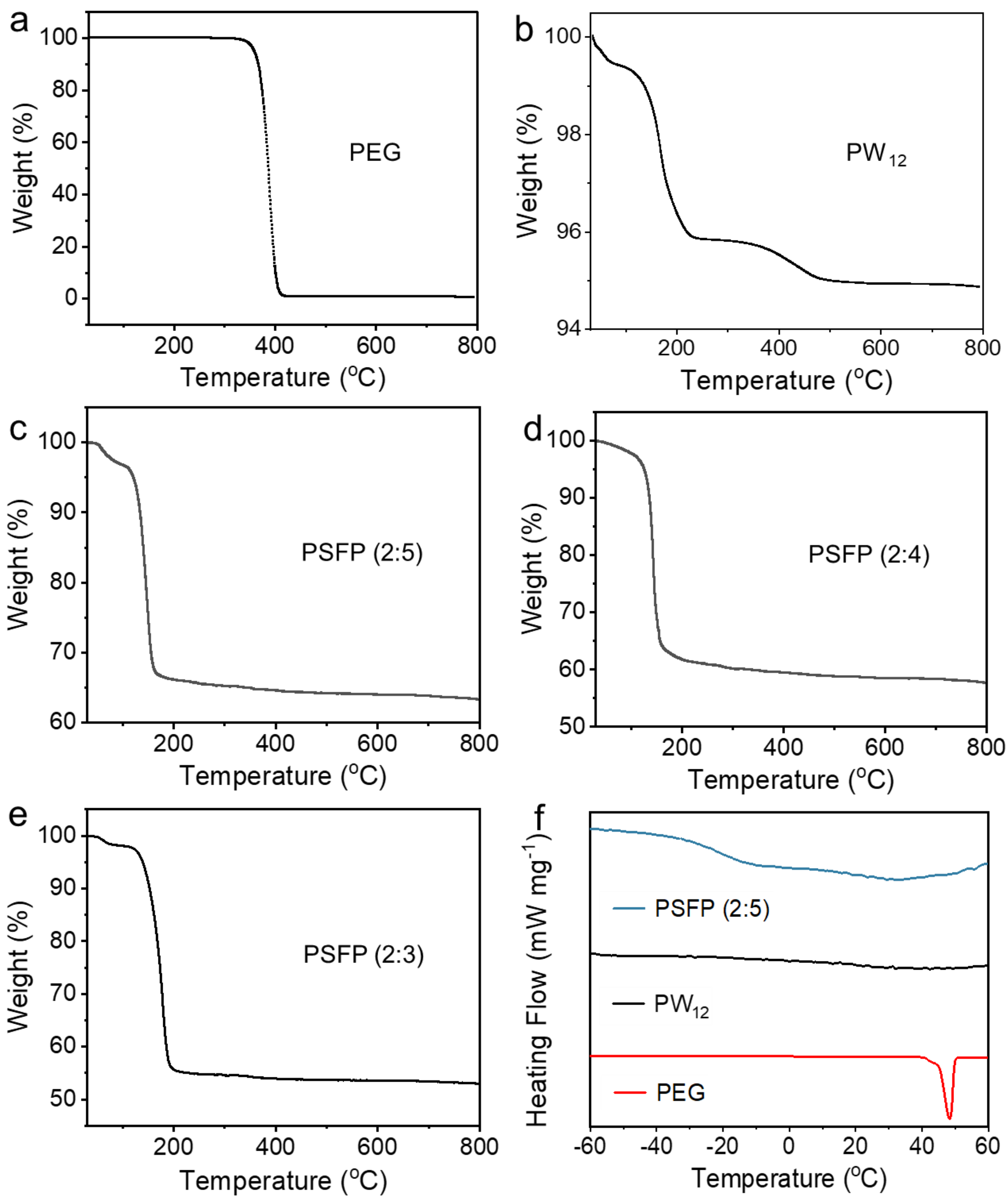

Supplementary Figure 23. TGA curves of the samples. a) PEG. b) PW_{12}. c-e) PSFP adhesives with different mass ratios (PEG : PW_{12} = 2 : 5, 2 : 4, and 2 : 3). f) DSC spectra of PSFP adhesive (2 : 5), PW_{12}, and PEG.

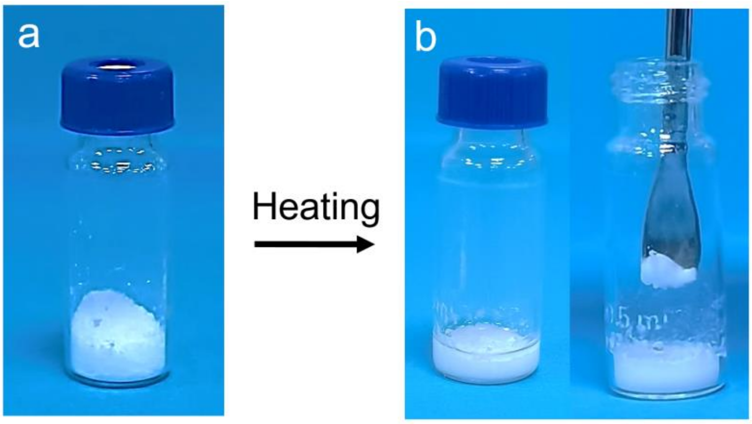

Supplementary Figure 24. Digital photographs of the samples. a) Mixture of PEG and $Na_3PW_{12}O_{40}$, b) Mixture of PEG and $Na_3PW_{12}O_{40}$ after heating at 90 ℃ for 2 h.

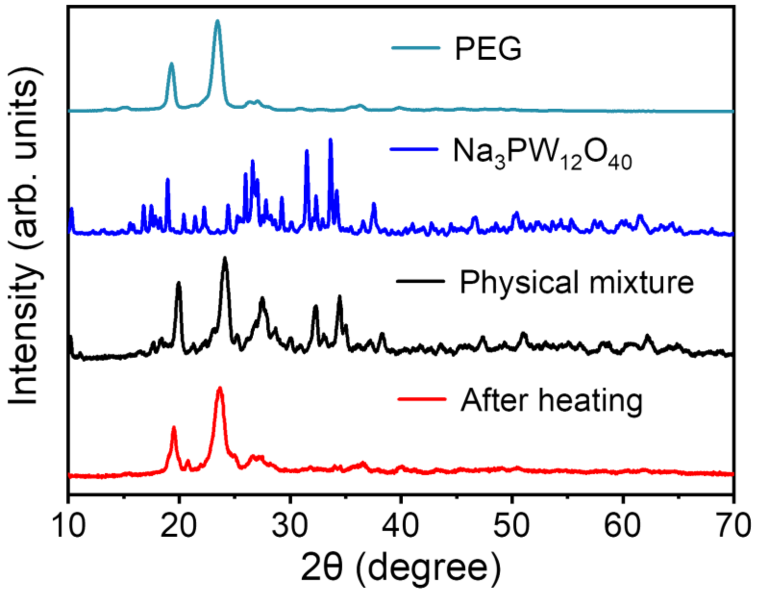

Supplementary Figure 25. PXRD patterns of the samples. a) PEG. b) $Na_3PW_{12}O_{40}$. c) Physical mixture of PEG and $Na_3PW_{12}O_{40}$. d) Mixture of PEG and $Na_3PW_{12}O_{40}$ after heating at 90 ℃ for 2 h.

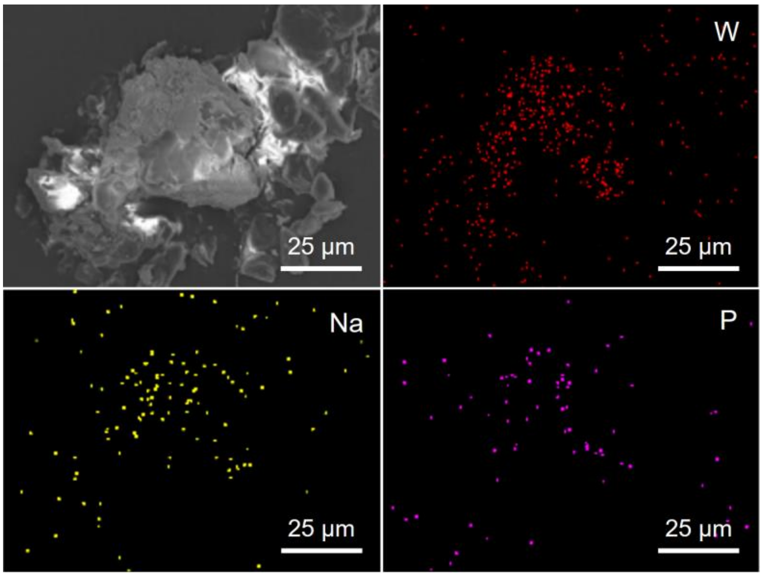

Supplementary Figure 26. SEM images of the mixture for $Na_3PW_{12}O_{40}$ and PEG after heating, and the corresponding elemental mapping.

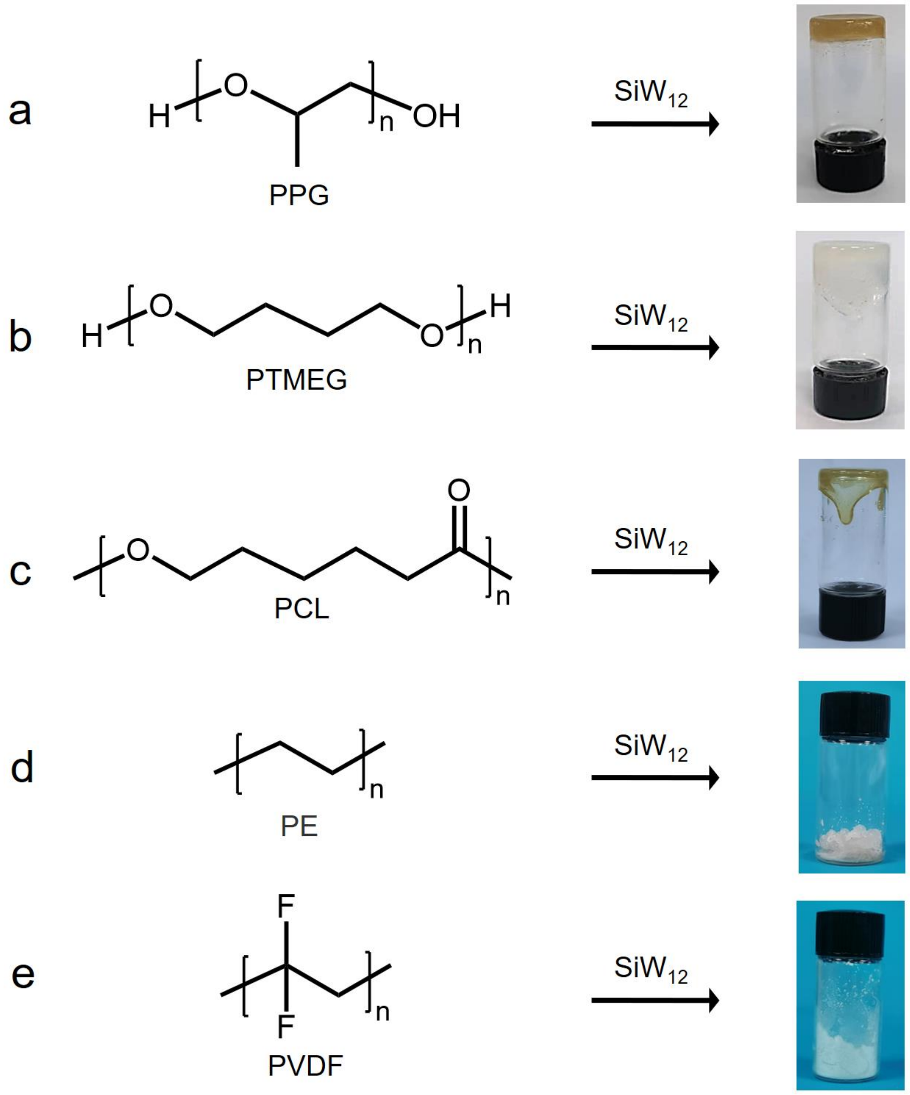

Supplementary Figure 27. Digital photographs of the samples after heating at 90 ℃ for 2 h. a) Adhesive based on PPG and SiW_{12}. b) Adhesive based on PTMEG and SiW_{12}. c) Adhesive based on PCL and SiW_{12}. d) Mixture of PE and SiW_{12}. e) Mixture of PVDF and SiW_{12}.

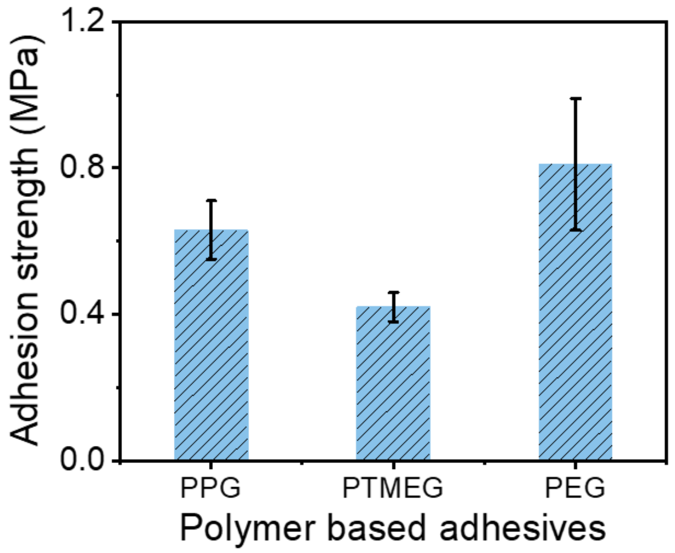

Supplementary Figure 28. Adhesion strengths of different polymer based adhesives on SS substrate: PPG, PTMEG and PEG (Mn, ~2000). The error bars represent mean ± standard deviation (n = 3 independent samples).

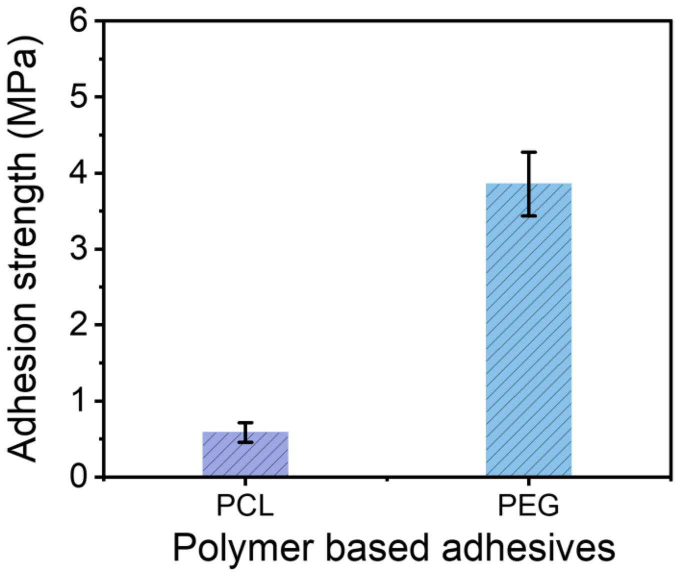

Supplementary Figure 29. Adhesion strengths of different polymer based adhesives on SS substrate: PCL and PEG (Mn, ~10000). The error bars represent mean ± standard deviation (n = 3 independent samples).

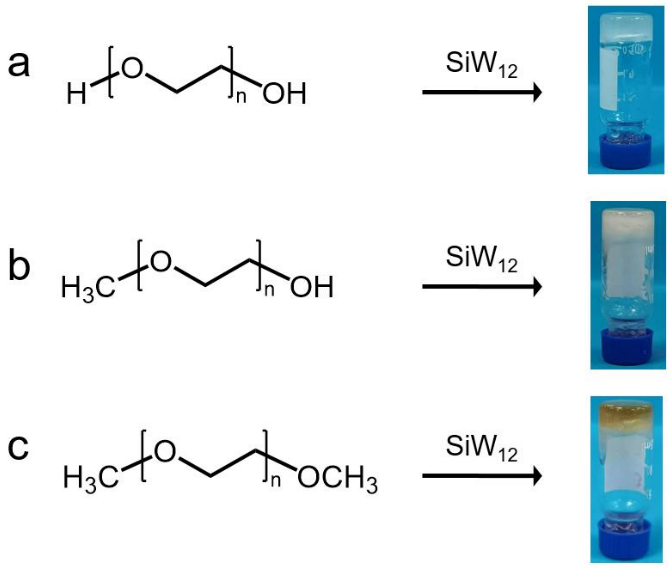

Supplementary Figure 30. Digital photographs of the PEG analogue based adhesives. a) PEG. b) PEGME. c) PEGdME (Mn, ~2000).

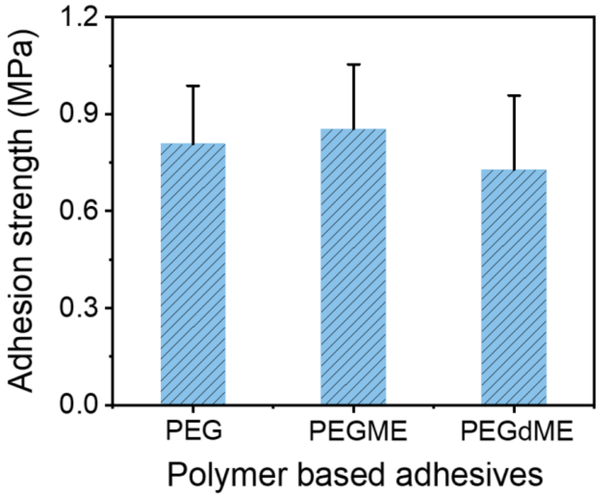

Supplementary Figure 31. Adhesion strengths of different polymer based adhesives on SS substrate: PEG, PEGME and PEGdME (Mn, ~2000). The error bars represent mean ± standard deviation (n = 3 independent samples).

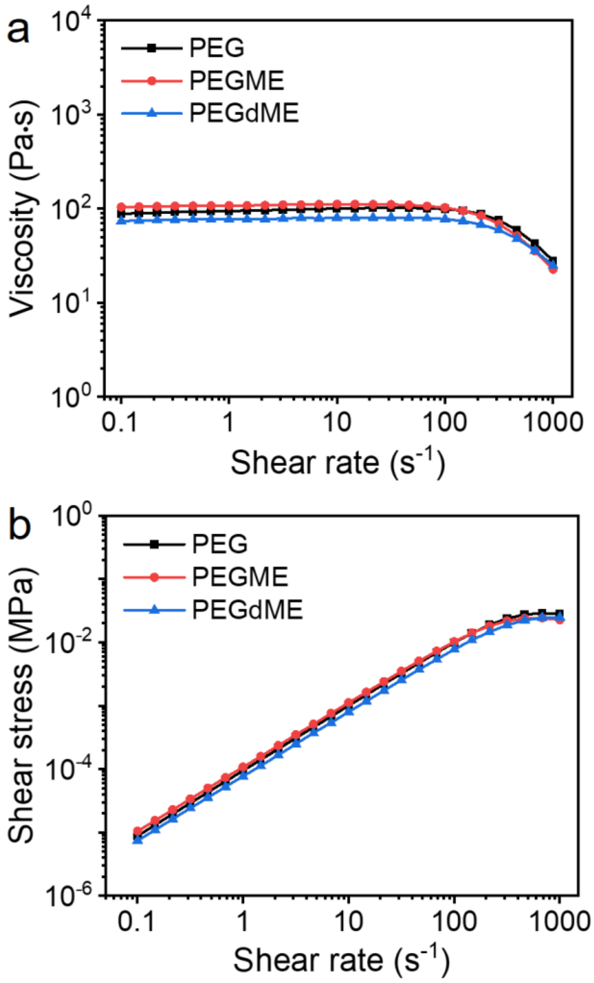

Supplementary Figure 32. Characterization of the different polymers based adhesives. a) Viscosity as a function of shear rate. b) Shear stress as a function of shear rate.

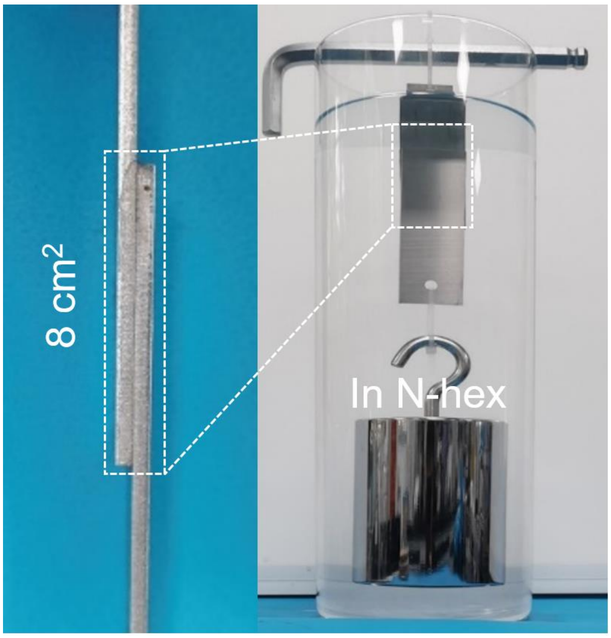

Supplementary Figure 33. Photographs of the SSFP adhesive after immersion in organic solvents (*N*-hex) for 14 days.

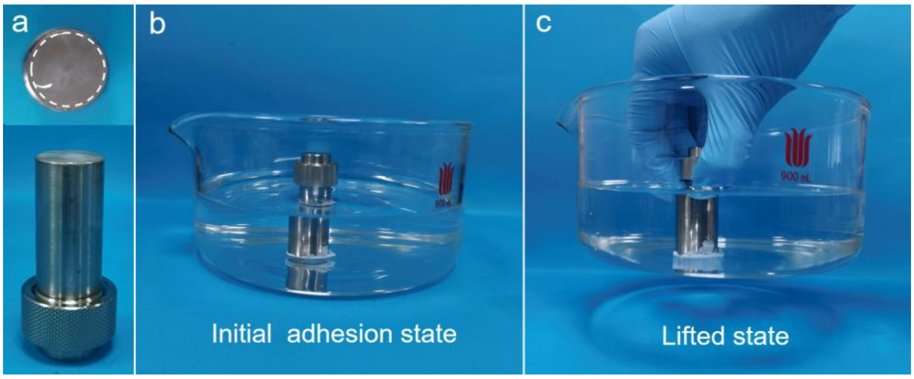

Supplementary Figure 34. Adhesion behavior of the SSFP adhesive in organic solvent. a) Stainless steel mould. b) Initial adhesion state. c) Lifted state (the adhesion area is 1.77 cm^2, and the volume of EA is 500 mL).

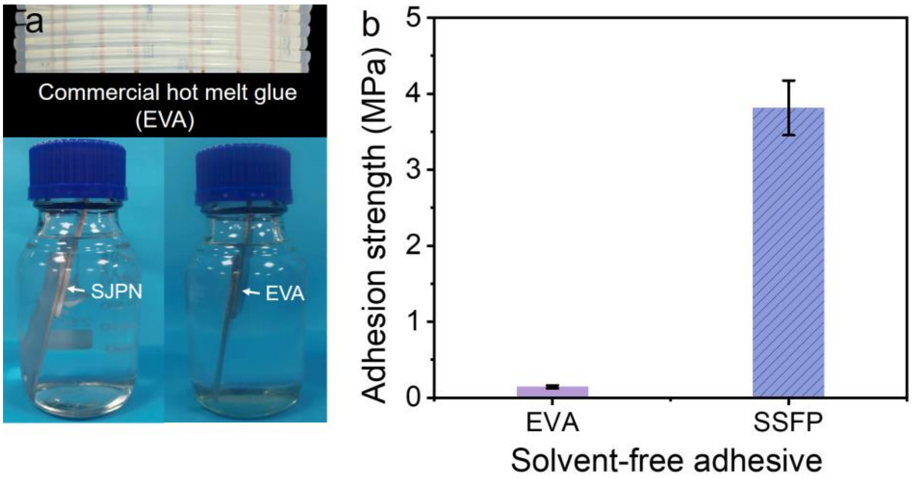

Supplementary Figure 35. Adhesion behavior of the solvent-free adhesives (EVA and SSFP). a) Photographs of the solvent-free adhesives soaking in mesitylene. b) Adhesion strength of the solvent-free adhesive adhered on SS substrate after soaking for 7 days. The error bars for **b** represent mean ± standard deviation (n = 3 independent samples).

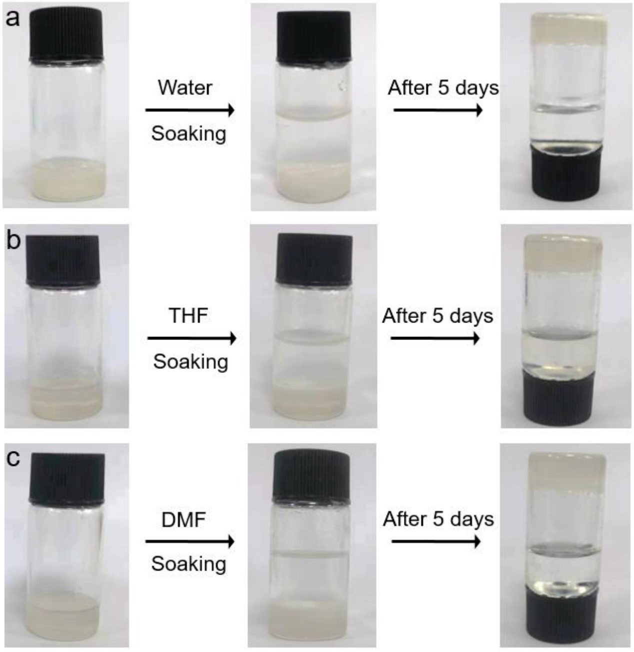

Supplementary Figure 36. Digital photographs of the adhesive after soaking in different solvents for 5 days. a) Water. b) THF. c) DMF.

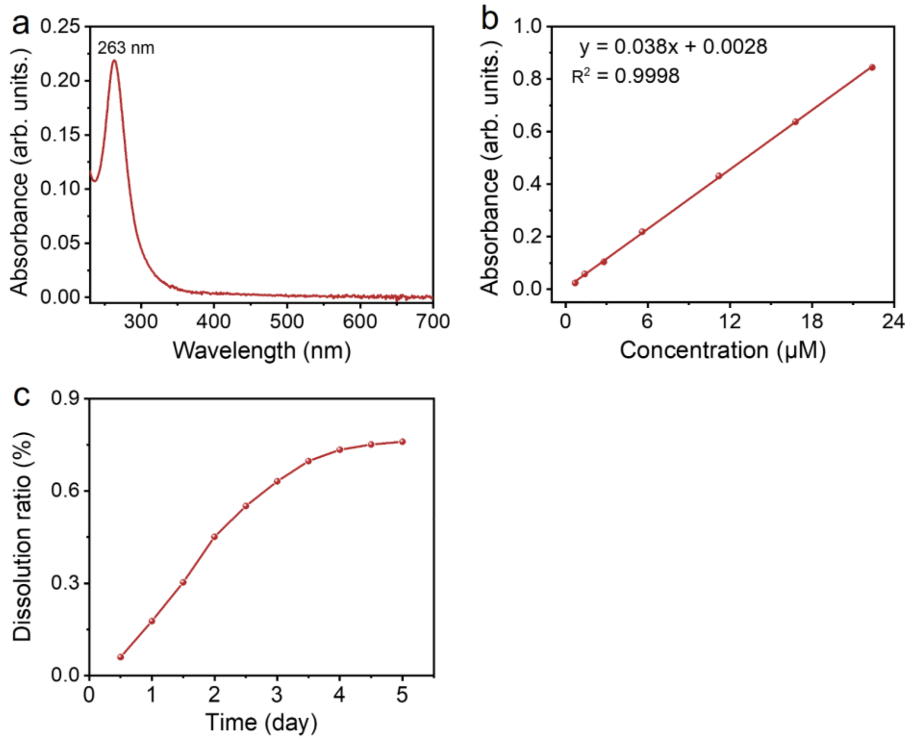

Supplementary Figure 37. The SiW_{12} leaching test of SSFP adhesive by soaking in water. a) UV-vis spectrum of SiW_{12} in water. b) The plot of absorbance change at 263 nm upon the concentration increase of SiW_{12}. c) The corresponding dissolution ratio of SiW_{12} in SSFP adhesive after soaking in water for different time.

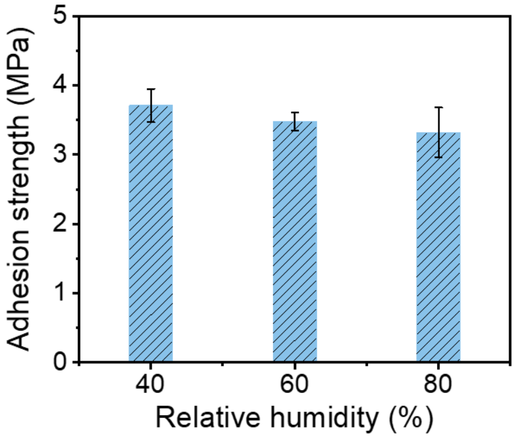

Supplementary Figure 38. Adhesion strengths of SSFP adhesive at different relative humidity. The error bars represent mean ± standard deviation (n = 3 independent samples).

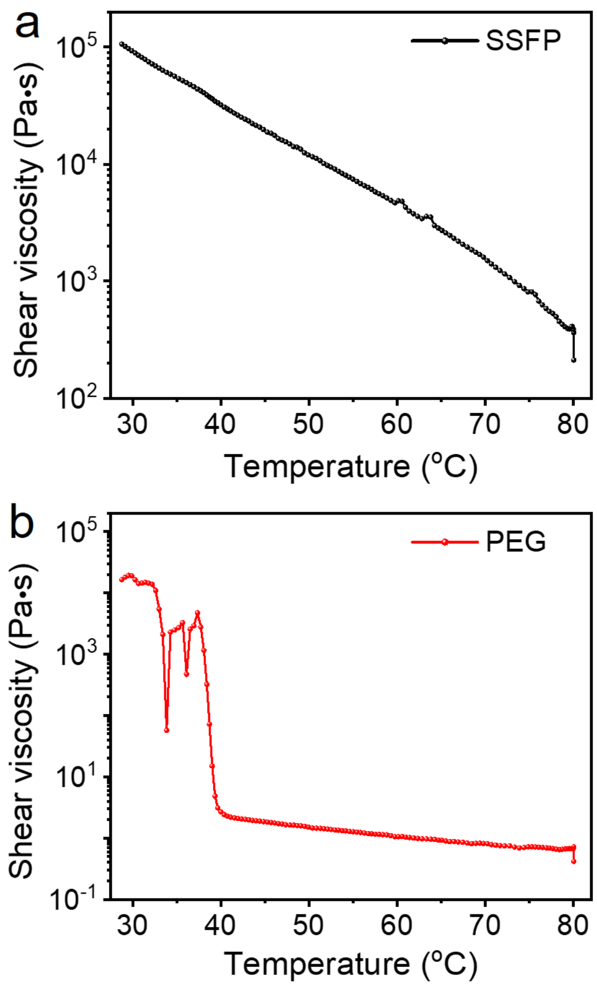

Supplementary Figure 39. Shear viscosity of the samples as a function of temperature. a) The SSFP adhesive. b) PEG.

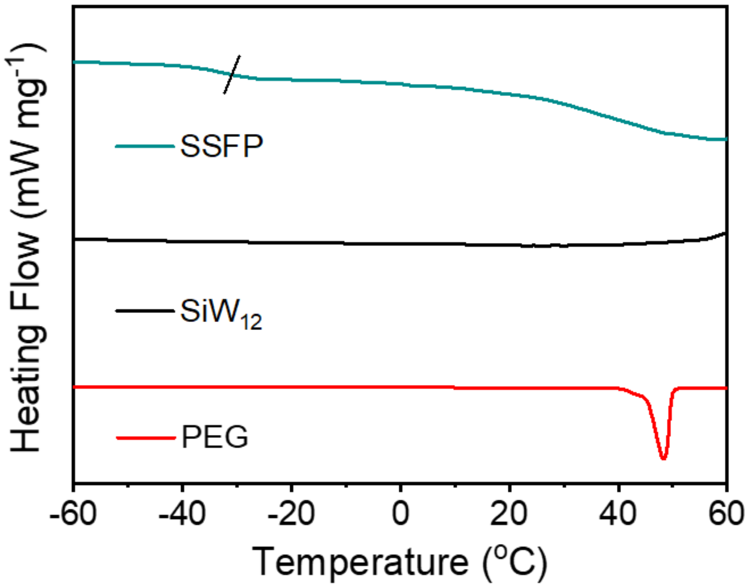

Supplementary Figure 40. DSC spectra of SSFP adhesive, SiW_{12}, and PEG.

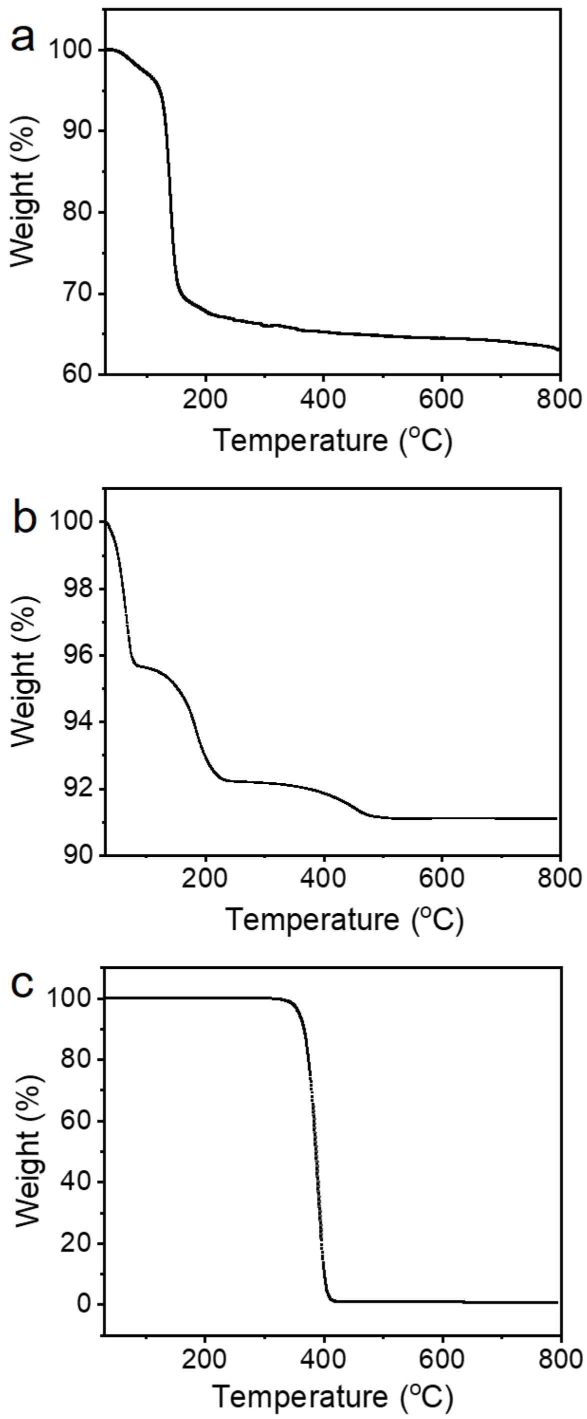

Supplementary Figure 41. TGA curves of SSFP adhesive, SiW_{12} and PEG. a) SSFP adhesive. b) SiW_{12}. c) PEG.

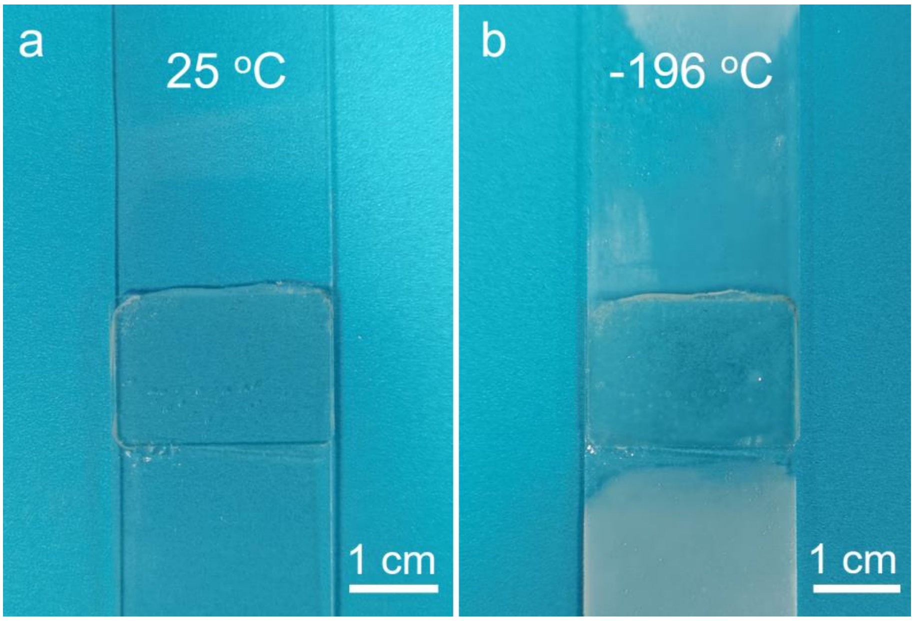

Supplementary Figure 42. Photographs of the SSFP adhesive adhered in glass slices after treatment. a) 25 ℃. b) -196 ℃.

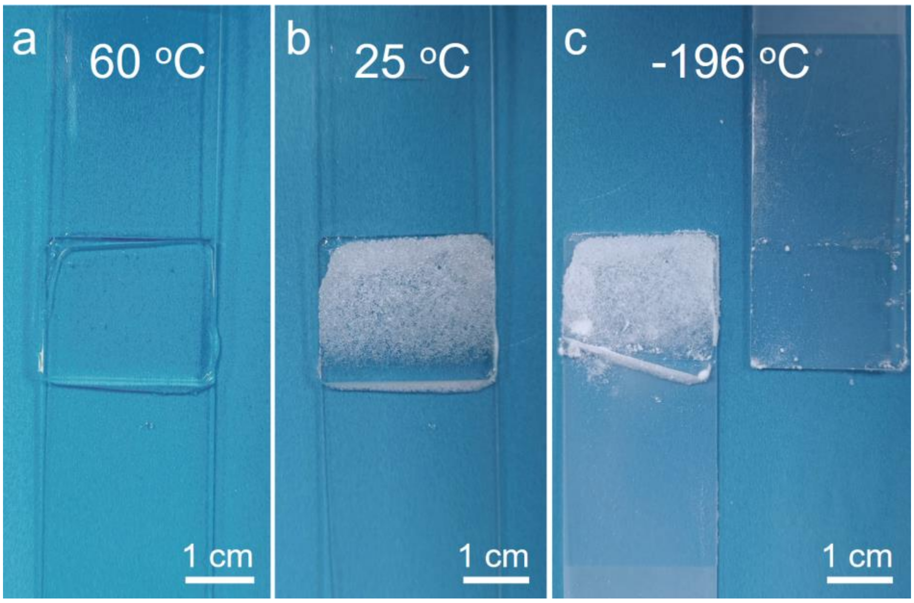

Supplementary Figure 43. Photographs of the PEG adhered in glass slices at different temperature. a) 60 ℃. b) 25 ℃. c) -196 ℃.

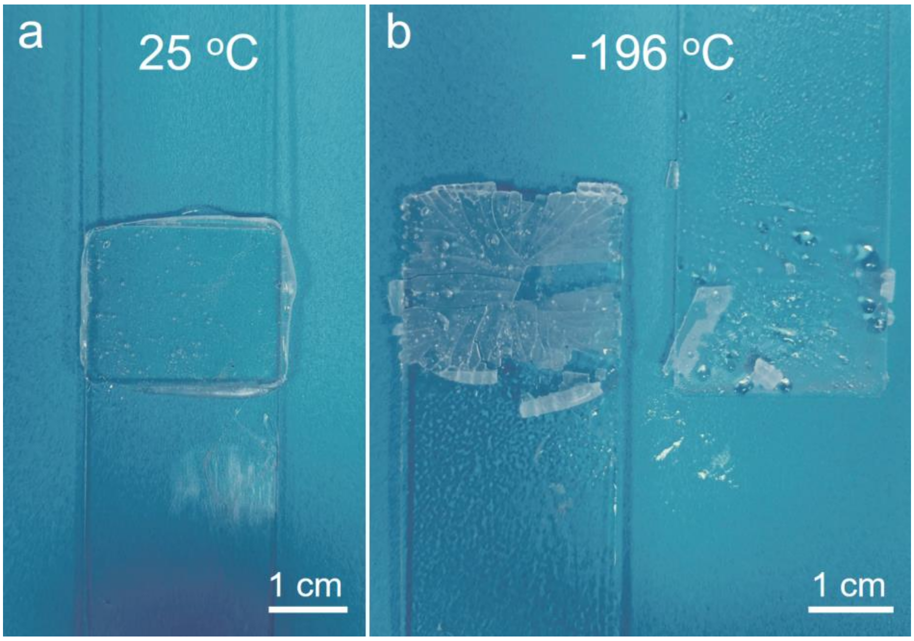

Supplementary Figure 44. Photographs of the EVA adhered in glass slices after treatment. a) 25 ℃. b) -196 ℃.

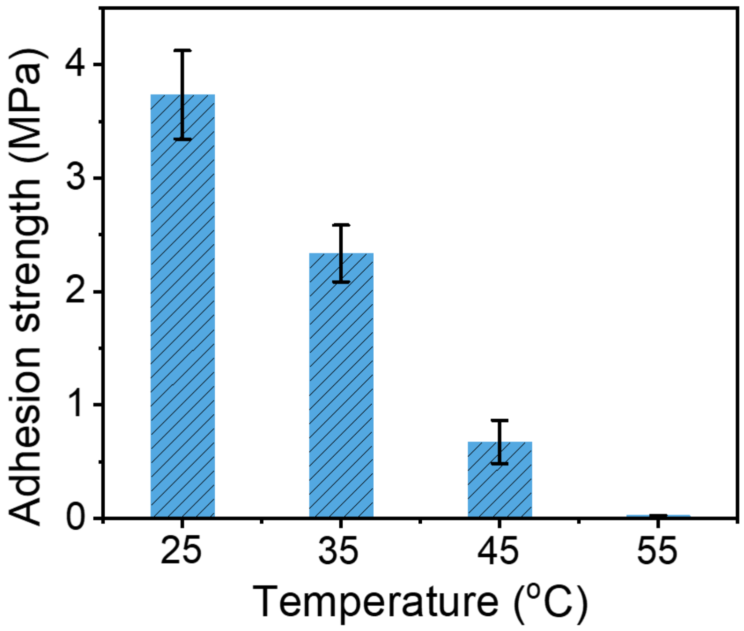

Supplementary Figure 45. Adhesion strengths of SSFP adhesive on SS substrate at various temperatures. The error bars represent mean ± standard deviation (n = 3 independent samples).

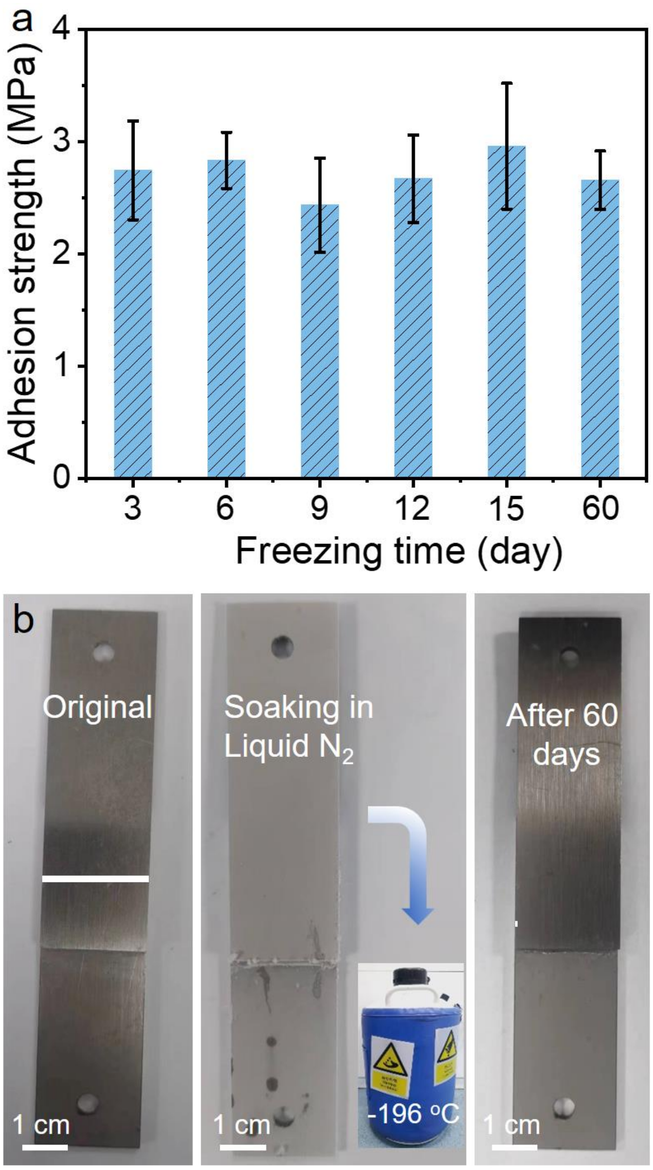

Supplementary Figure 46. Adhesion performance of SSFP adhesive in liquid nitrogen (-196 ℃). a) Adhesion strength of SSFP adhesive frozen for different times. b) The photograph of SSFP adhesive on SS after soaking in liquid nitrogen (-196 ℃) for 60 days. The error bars for **a** represent mean ± standard deviation (n = 3 independent samples).

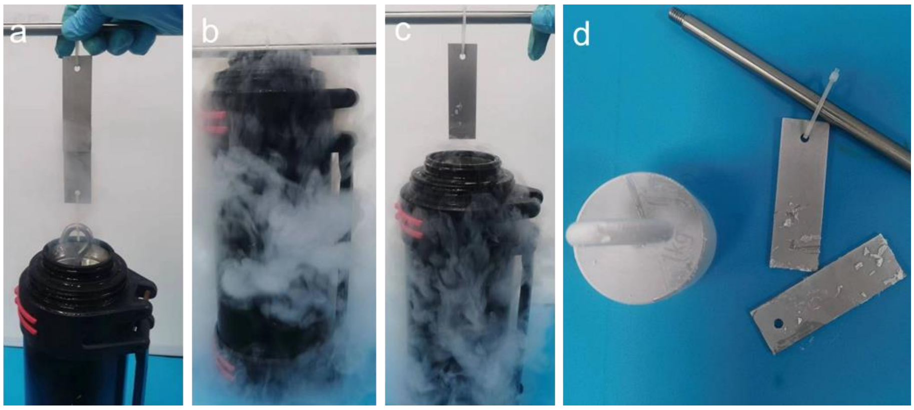

Supplementary Figure 47. Adhesion tests of the commercial hot melt glue EVA in liquid nitrogen. a) Initial adhesion state. b) Immersed state. c-d) Frost-cracked state.

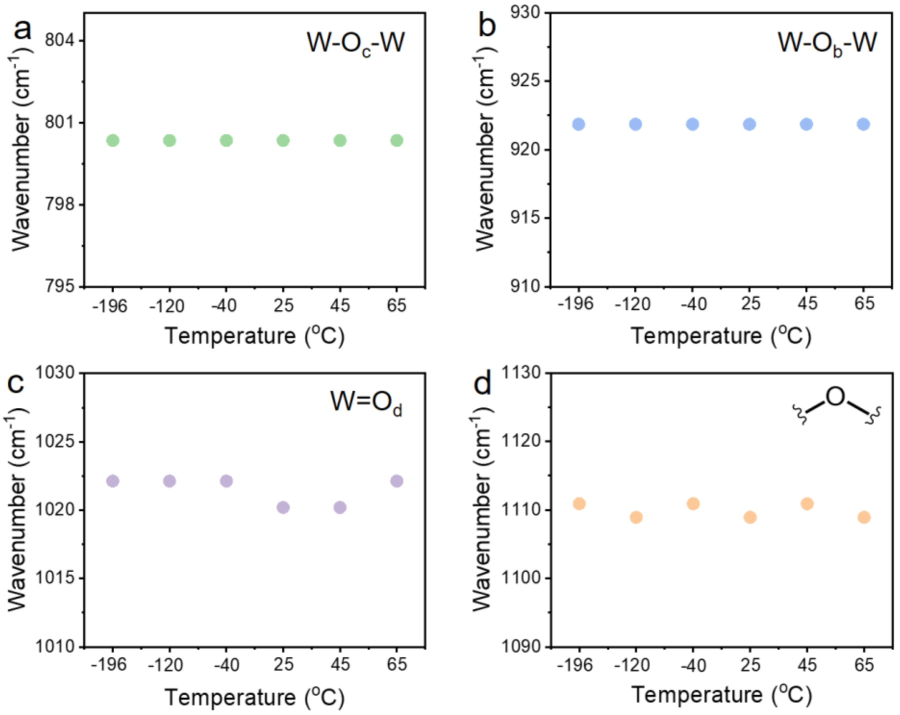

Supplementary Figure 48. Temperature-dependent FT-IR spectra of SSFP adhesive at different temperature conditions. a) W–O_c–W. b) W–O_b–W. c) W=O_d. d) C–O–C.

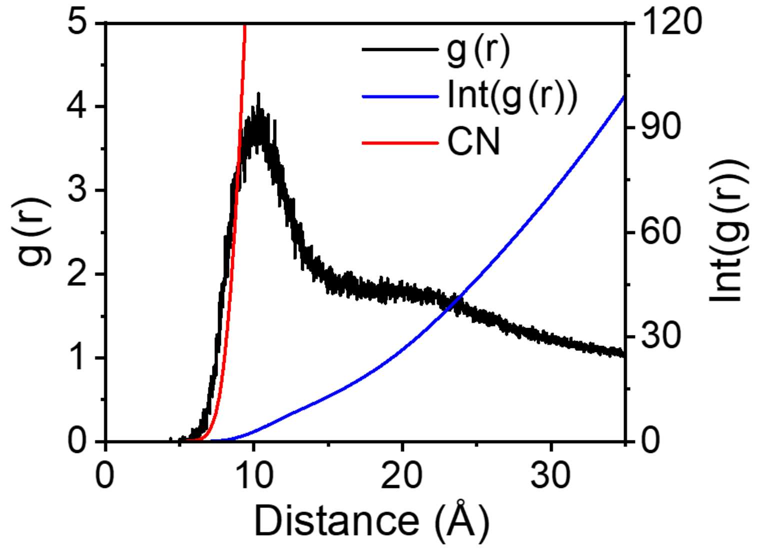

Supplementary Figure 49. The radial distribution function (RDF) of POMs and PEGs for the production of 1 ns.

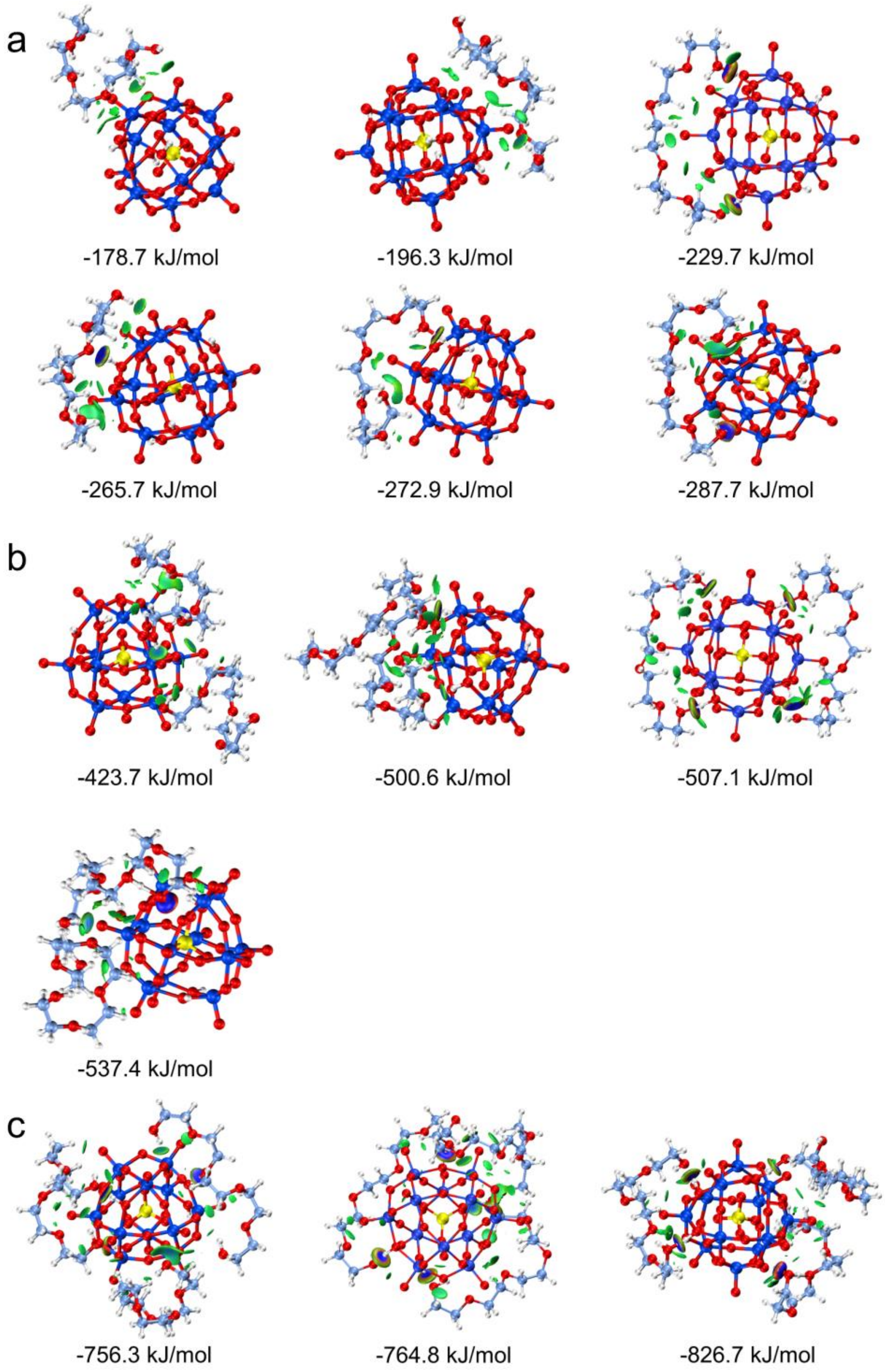

Supplementary Figure 50. Independent gradient model based on Hirshfeld partition (IGMH) and interaction energies (ΔE, kJ/mol) between SiW_{12} and PEG for the adhesive formation. a) SiW_{12} and one PEGs. b) SiW_{12} and two PEGs. c) SiW_{12} and three PEGs.

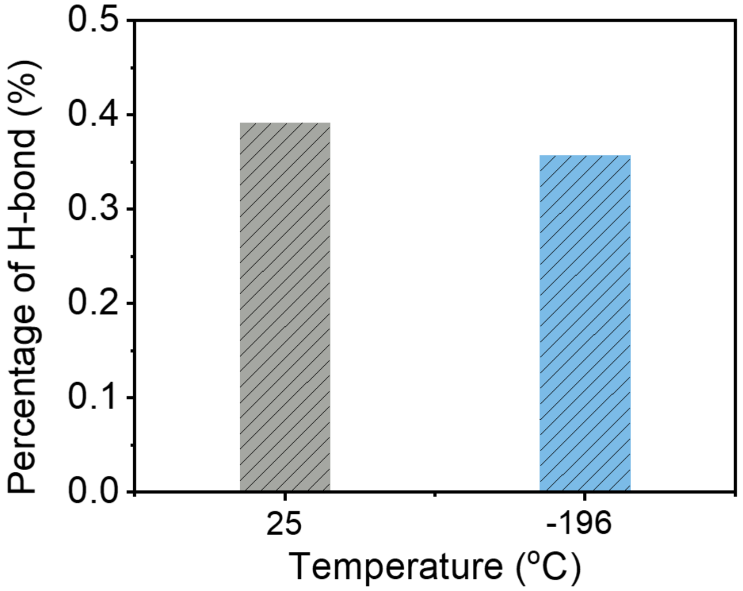

Supplementary Figure 51. The ratio of formative hydrogen bonds between PEG and SiW_{12} at 25 and -196 ℃ for the final 2 ns of the NVT simulation.

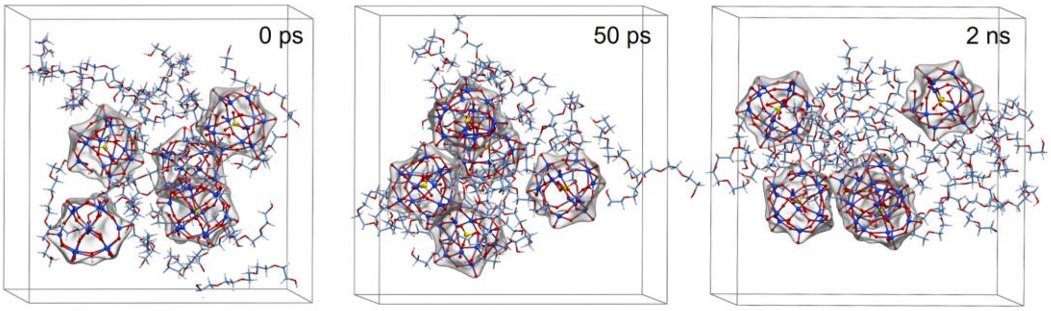

Supplementary Figure 52. Snapshots of the aggregation behavior of PEG and SiW_{12} at 55 ℃ (the cubic boundaries are marked with black lines).

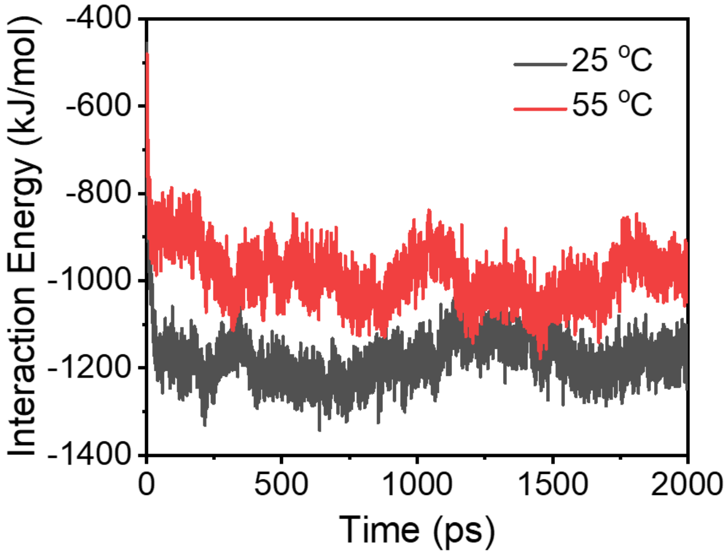

Supplementary Figure 53. The interaction energy of PEG and SiW_{12} during cross-linking process at 25 and 55 ℃ for 2 ns of the NVT simulation.

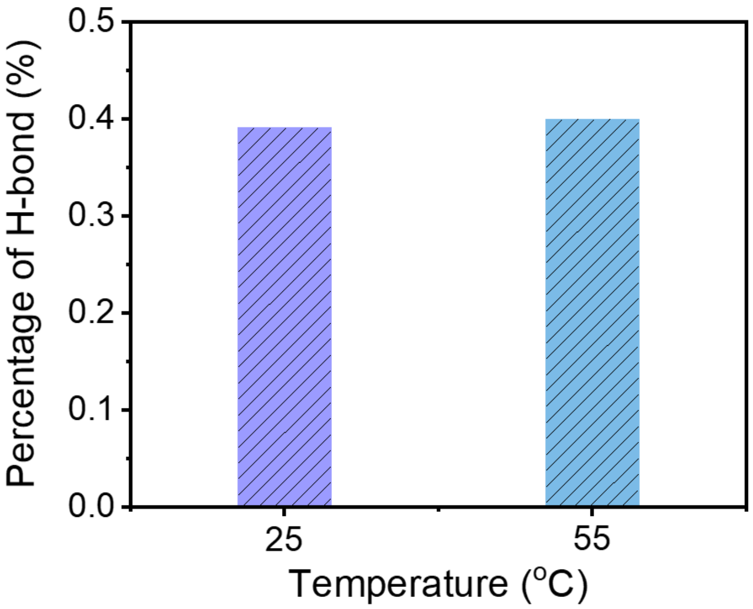

Supplementary Figure 54. The ratio of formative hydrogen bonds between PEG and SiW_{12} at 25 and 55 ℃ for the final 2 ns of the NVT simulation.

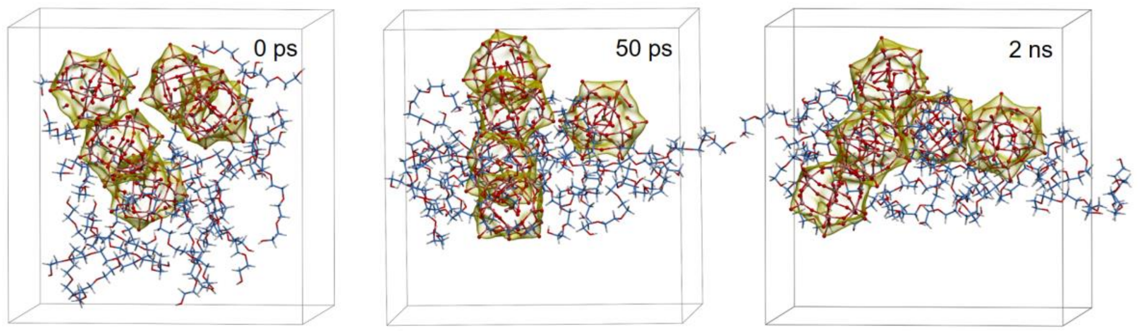

Supplementary Figure 55. Snapshots of the aggregation behavior of PEG and PW_{12} at 25 ℃ (the cubic boundaries are marked with black lines).

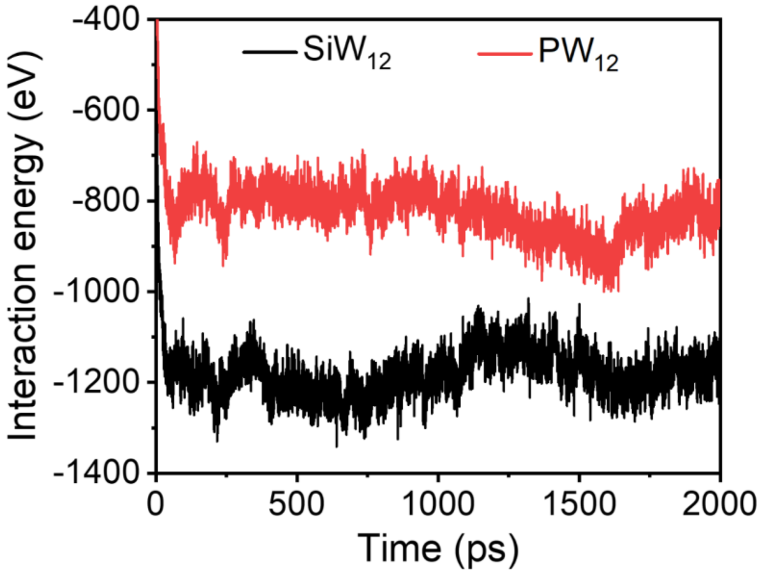

Supplementary Figure 56. The interaction energy of PEG and POMs (SiW_{12} and PW_{12}) during cross-linking process at 25 ℃ for 2 ns of the NVT simulation.

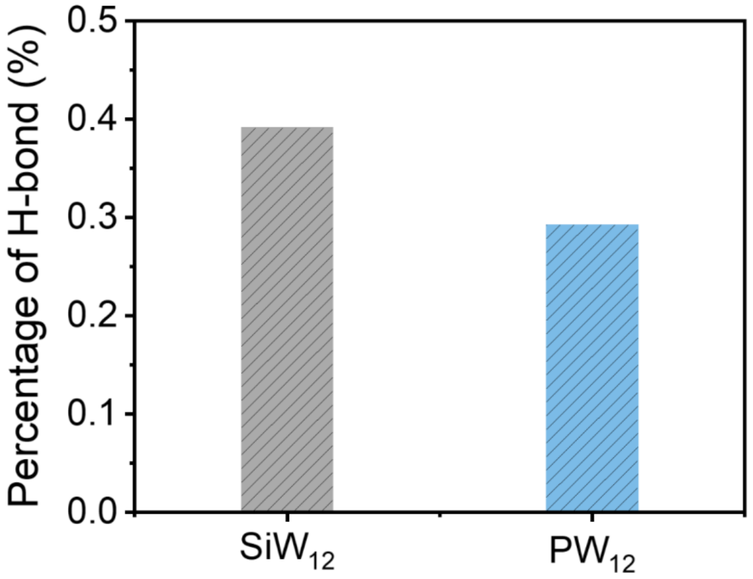

Supplementary Figure 57. The ratio of formative hydrogen bonds between PEG and POMs (SiW_{12} and PW_{12}) at 25 ℃ for the final 2 ns of the NVT simulation.

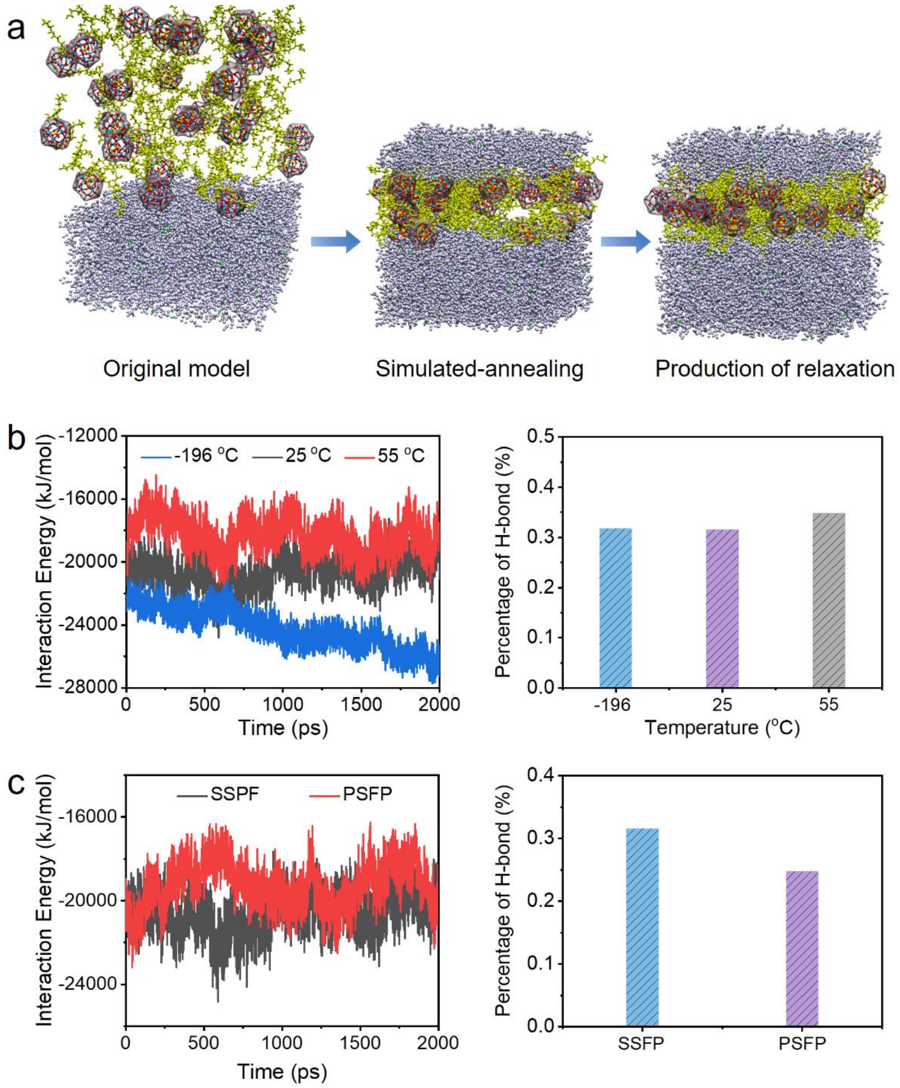

Supplementary Figure 58. Adhesive mechanism and MD simulation based on the NPT ensemble. a) MD simulations of configurations of molecular models of SSFP and SS substrate. b) The interaction energy and the ratio of formative hydrogen bonds between SSFP adhesive and SS substrate at-196, 25, and 55 ℃. c) The comparison of interaction energy and the ratio of formative hydrogen bonds between SSFP and PSFP adhesive on SS substrate.

Supplementary Table

Supplementary Table 1. Performance comparison of the reported POMs based adhesives, and the test method is the same in the all references (lap joint).

Sample	POM	Cross-linked compound	Type	Temperature (°C)	Substrate	Adhesion strength (kPa)	Ref.
**SSFP**	**SiW_{12}**	**PEG (10 kDa)**	**Solvent-free**	**-196<br>RT**	**SS**	**2741.0<br>3736.0**	**This work**
POMs/Pep	BW_{12}/<br>$PMo_{11}V$	Ac-KKNSQCC-NH_2/GHK	Solvent-assisted (H_2O)	RT	Ti	82.5	12
SiW-PEG	SiW_{12}	PEG (20 kDa)	Solvent-assisted (H_2O)	RT	SS	74.2	13
Pep1/SiW_{11}/H^+<br>Pep1/SiW_{11}/M^{n+}	SiW_{11}	GHK/H^+<br>GHK/Co^{2+}	Solvent-assisted (H_2O)	RT	Ti	36.5<br>21.1	14
P-PA55-PW30	PW_{12}	Polyacrylate (100 kDa)/H_3PO_4	Solvent-assisted (CH_3COCH_3)	RT	Glass	211.0	15
PEI-PW_{12}	PW_{12}	PEI (1.8 kDa)	Solvent-assisted (H_2O)	RT	Wood	319.0	16
Ca-POM SNWs	PW_{12}	Oleylamine/Ca^{2+}	Solvent-assisted (H_2O)	-196<br>100	SS	2160.0<br>~ 1700.0	17
Pep1/SiW	SiW_{12}	Ac-EEMQRRAD-NH_2	Solvent-assisted (H_2O)	RT	PEEK	29.6	18
GSSG/HPW	PW_{12}	GSSH	Solvent-assisted (H_2O)	RT	Ti	53.3	19
NA/HP_2W_{18}	P_2W_{18}	3-(2-naphthyl)-l-alanine	Solvent-assisted (H_2O)	RT	PEEK	14.7	20
His/SiW	SiW_{12}	Histidine	Solvent-assisted (H_2O)	RT	Wood	436.7	21
CS/SiW-PAM	SiW_{12}	Polyacrylamide/ Chitosan (3 kDa)	Solvent-assisted (H_2O)	RT	PP	7.0	22

Supplementary Table 2. Comparison of representative solvent-free adhesives in terms of their adhesion strength, low temperature tolerance and temperature tolerance range.

**Sample**	**Adhesion strengths (MPa)**	**Low temperature tolerance (MPa)**	**Temperature tolerance range (°C)**	**Ref.**
**SSFP**	**3.70**	**2.96 (-196 °C)**	**-196-55**	**This work**
SEA0.2	10.2	N/A	N/A	23
DESPs	6.57	1.30 (-80 °C)	-80-80	24
Poly(TtADO-TA)-2	4.55	N/A	N/A	25
CT-2	4.40	> 1.0 (-80 °C)	N/A	26
Poly(TA-DIB-Fe) 50:1	2.50	N/A	0-60	27
PC10−W1	2.49	1.17 (-196 °C)	-196-25	28
Ca-POM SNWs	2.16	1.70 (-196 °C)	-196-100	17
6-HTPB	2.14	0.50 (-18 °C)	-80-80	29
Pt-B21C7-II	1.90	N/A	N/A	30
DPETI	1.84	2.22 (-196 °C)	N/A	31
AZO-P1	1.34	N/A	25-50	32
CA/PEG_{2000}	0.53	0.85 (-196 °C)	-196-25	33
TADP30	0.36	N/A	25-50	34
P-PA55-PW30	0.21	N/A	N/A	35
LTFe	0.02	N/A	N/A	36

Supplementary References

1. Frisch, M. J. *et al.* Gaussian 09, Revision D.01, Gaussian Inc., Wallingford CT. (2019).
2. Grimme, S., Antony, J., Ehrlich, S. & Krieg, H. A consistent and accurate ab initio parametrization of density functional dispersion correction (DFT-D) for the 94 elements H-Pu. *J. Chem. Phys.* **132**, 154104 (2010).
3. Marenich, A. V., Cramer, C. J. & Truhlar, D. G. Universal solvation model based on solute electron density and on a continuum model of the solvent defined by the bulk dielectric constant and atomic surface tensions. *J. Phys. Chem. B* **113**, 6378-6396 (2009).
4. Lu, T. & Chen, F. Multiwfn: A multifunctional wavefunction analyzer. *J. Comput. Chem.* **33**, 580-592 (2012).
5. Humphrey, W., Dalke, A. & Schulten K. VMD: Visual molecular dynamics. *J. Mol. Graph.* **14**, 33-38 (1996).
6. Berendsen, H. J. C., van der Spoel, D. & van Drunen, R. GROMACS: A message-passing parallel molecular dynamics implementation. *Comp. Phys. Comm.* **91**, 43-56 (1995).
7. van der Spoel, D. *et al.* GROMACS: fast, flexible and free. *J. Comp. Chem*. **26**, 1701-1718 (2005.)
8. Hess, B., Kutzner, C., van der Spoel, D. & Lindahl, E. GROMACS 4: algorithms for highly efficient, load-balanced, and scalable molecular simulation. *J. Chem. Theory Comput.* **4**, 435-447 (2008).
9. Jorgensen, W. L., Madura, J. D. & Swenson, C. J. Optimized intermolecular potential functions for liquid hydrocarbons. *J. Am. Chem. Soc*. **106**, 6638-6646 (1984).
10. Rappé, A. K., Casewit, C. J., Colwell, K. S., Goddard III, W. A. & Skiff, W. M. UFF, a full periodic table force field for molecular mechanics and molecular dynamics simulations. *J. Am. Chem. Soc.* **114**, 10024-10035 (1992).
11. Berendsen, H. J. C., Postma, J. P. M., Vangunsteren, W. F., Dinola, A. & Haak, J. R. *J. Chem. Phys.* **81**, 3684-3690 (1984).
12. Liu, X., Ma, Z., Nie, J., Fang, J. & Li, W. Exploiting redox-complementary peptide/polyoxometalate coacervates for spontaneously curing into antimicrobial adhesives. *Biomacromolecules* **23**, 1009-1019 (2022).
13. Peng, Q. *et al.* Adhesive coacervates driven by hydrogen-bonding interaction. *Small* **16**, e2004132 (2020).
14. Li, X. *et al.* Coassembly of short peptide and polyoxometalate into complex coacervate adapted for pH and metal ion-triggered underwater adhesion. *Langmuir* **35**, 4995-5003 (2019).
15. Guo, H. *et al.* Multifunctional enhancement of proton-conductive, stretchable, and adhesive performance in hybrid polymer electrolytes by polyoxometalate nanoclusters. *ACS Appl. Mater. Interfaces* **13**, 30039-30050 (2021).
16. Cui, Y. *et al.* A universal and reversible wet adhesive via straightforward aqueous self-assembly of polyethylenimine and polyoxometalate. *ACS Appl. Mater. Interfaces* **13**, 47155-47162 (2021).
17. Zhang, S., Shi, W., Yu, B. & Wang, X. Versatile inorganic subnanometer nanowire adhesive. *J. Am. Chem. Soc.* **144**, 16389-16394 (2022).

18. Xu, J. *et al.* Supramolecular copolymerization of short peptides and polyoxometalates: toward the fabrication of underwater adhesives. *Biomacromolecules* **18**, 3524-3530 (2017).
19. Liu, X. *et al.* Heteropoly acid-driven assembly of glutathione into redox-responsive underwater adhesive. *Chem. Commun.* **56**, 11034-11037 (2020).
20. Li, X. *et al.* Bringing heteropolyacid-based underwater adhesive as printable cathode coating for self-powered electrochromic aqueous batteries. *Adv. Funct. Mater.* **28**, (2018).
21. Xu, J. *et al.* Wet and functional adhesives from one-step aqueous self-assembly of natural amino acids and polyoxometalates. *Angew. Chem. Int. Ed.* **56**, 8731-8735 (2017).
22. Wei, X. *et al.* Adhesive, conductive, self-healing, and antibacterial hydrogel based on chitosan–polyoxometalate complexes for wearable strain sensor. *ACS Appl. Polym. Mater.* **2**, 2541-2549 (2020).
23. Sun, P., Li, Y., Qin, B., Xu, J.-F. & Zhang, Xi. Super strong and multi-reusable supramolecular epoxy hot melt adhesives. *ACS Materials Lett.* **3**, 1003-1009 (2021).
24. Wu, S., Cai, C., Li, F., Tan, Z. & Dong, S. Deep eutectic supramolecular polymers: new type of bulk supramolecular materials. *Angew. Chem. Int. Ed.* **59**, 11871-11875 (2020).
25. Yang, S., Bai, J., Sun, X. & Zhang, J. Robust and healable poly(disulfides) supramolecular adhesives enabled by dynamic covalent adaptable networks and noncovalent hydrogen-bonding interactions. *Chem. Eng. J.* **461,** 142066 (2023).
26. Wu, S., Cai, C., Li, F., Tan, Z. & Dong, S. Supramolecular adhesive materials from natural acids and sugars with tough and organic solvent-resistant adhesion. *CCS Chem.* **2**, 1690-1700 (2020).
27. Zhang, Q., Shi, C.-Y., Qu, D.-H., Long, Y.-T., Feringa, B. L. & Tian, H. Exploring a naturally tailored small molecule for stretchable, self-healing, and adhesive supramolecular polymers. *Sci. Adv.* **4**, eaat8192 (2018).
28. Li, X., Lai, J., Deng, Y., Song, J., Zhao, Gai. & Dong, S. Supramolecular adhesion at extremely low temperatures: a combined experimental and theoretical investigation. *J. Am. Chem. Soc.* **142**, 21522-21529 (2020).
29. M., Li. *et al.* Recoverable solvent-free small molecular supramolecular pseudoeutectic adhesives with a wide temperature range. *Green Chem.* **25**, 6845-6852 (2023).
30. Zhang, Q. *et al.* Formation of a supramolecular polymeric adhesive via water-participant hydrogen bond formation. *J. Am. Chem. Soc.* **141**, 8058-8063 (2019).
31. Chen, H. *et al.* Upcycling waste thermosetting polyimide resins into high-performance and sustainable low-temperature-resistance adhesives. *Adv. Mater.* **36**, 2310779 (2024).
32. Wu, Z. *et al.* Green-light-triggered phase transition of azobenzene derivatives toward reversible adhesives. *J. Am. Chem. Soc.* **141**, 7385-7390 (2019).
33. Xie, X., Xu, X. & Jiang, Y. Hydrogen-bonding interaction-driven catechin assembly into solvent-free supramolecular adhesive with antidrying and antifreezing properties. *ACS Appl. Polym. Mater.* **4**, 4319-4328 (2022).
34. Chen, J., Guo, D., Liang, S. & Liu, Z. A supramolecular copolymer based on small molecules, used for a multifunctional adhesive and rapid hemostasis. *Polym. Chem.* **11**, 6670-6680 (2020).
35. Guo, H. et al., Semi-solid superprotonic supramolecular polymer electrolytes based on deep

eutectic solvents and polyoxometalates. *Angew. Chem. Int. Ed.* **61**, e202210695 (2022).

36. Ke, X., et al., An instant, repeatable and universal supramolecular adhesive based on natural small molecules for dry/wet environments. *Chem. Eng. J.* **442**, 136206 (2022).
