# Supplementary material for: A solvent-free processed low-temperature tolerant adhesive
Source: Nat Commun. 2024 Jun 12;15:5017. doi: 10.1038/s41467-024-49503-7 (PMC11169673; doi:10.1038/s41467-024-49503-7)
Supplement: Supplementary file 3 — Description of Additional Supplementary Files [file 41467_2024_49503_MOESM3_ESM.pdf]

Description of Additional Supplementary Files

File Name: Supplementary Movie 1
Description: The SSFP adhesive performing rapid adhesion in organic solvent.

File Name: Supplementary Movie 2
Description: The SSFP adhesive preventing an emergency leakage for organic solvent.

File Name: Supplementary Movie 3
Description: The SSFP adhesive performing low-temperature resistance.

File Name: Supplementary Movie 4
Description: The MD of the SSFP adhesive at 25 ℃.

File Name: Supplementary Movie 5
Description: The MD of the SSFP adhesive at 55 ℃.

File Name: Supplementary Movie 6
Description: The MD of the SSFP adhesive at -196 ℃.

File Name: Supplementary Movie 7
Description: The MD of SSFP adhesive and SS substrate at 25 ℃.

File Name: Supplementary Movie 8
Description: The MD of SSFP adhesive and SS substrate at 55 ℃.

File Name: Supplementary Movie 9
Description: The MD of SSFP adhesive and SS substrate at -196 ℃.
